# Ti_3_C_2_T_x_ MXene‐Decorated 3D‐Printed Ceramic Scaffolds for Enhancing Osteogenesis by Spatiotemporally Orchestrating Inflammatory and Bone Repair Responses

**DOI:** 10.1002/advs.202400229

**Published:** 2024-07-08

**Authors:** Benzhao Huang, Shishuo Li, Shimin Dai, Xiaoqing Lu, Peng Wang, Xiao Li, Zhibo Zhao, Qian Wang, Ningbo Li, Jie Wen, Yifang Liu, Xin Wang, Zhentao Man, Wei Li, Bing Liu

**Affiliations:** ^1^ Department of Stomatology Shandong Provincial Hospital Affiliated to Shandong First Medical University Jinan Shandong 250021 P. R. China; ^2^ Department of Joint Surgery Shandong Provincial Hospital Affiliated to Shandong First Medical University Jinan Shandong 250021 P. R. China; ^3^ Medical Science and Technology Innovation Center Shandong First Medical University & Shandong Academy of Medical Sciences Jinan Shandong 250117 P. R. China; ^4^ College of Sports Medicine and Rehabilitation Shandong First Medical University & Shandong Academy of Medical Sciences Jinan Shandong 250117 P. R. China; ^5^ College of Engineering and Applied Sciences National Laboratory of Solid State Microstructure Collaborative Innovation Center of Advanced Microstructures Nanjing University Nanjing 210023 P. R. China; ^6^ School of Stomatology Shandong First Medical University & Shandong Academy of Medical Sciences Jinan Shandong 250021 P. R. China; ^7^ Endocrine and Metabolic Diseases Hospital of Shandong First Medical University Shandong Institute of Endocrine and Metabolic Diseases Jinan Shandong 250062 P. R. China

**Keywords:** 3D‐printed scaffolds, Mxene, zinc/strontium ion, near‐infrared responsivity, spatiotemporal regulation of inflammatory

## Abstract

Inflammatory responses play a central role in coordinating biomaterial‐mediated tissue regeneration. However, precise modulation of dynamic variations in microenvironmental inflammation post‐implantation remains challenging. In this study, the traditional β‐tricalcium phosphate‐based scaffold is remodeled via ultrathin MXene‐Ti_3_C_2_ decoration and Zn^2+^/Sr^2+^ ion‐substitution, endowing the scaffold with excellent reactive oxygen species‐scavenging ability, near‐infrared responsivity, and enhanced mechanical properties. The induction of mild hyperthermia around the implant via periodic near‐infrared irradiation facilitates spatiotemporal regulation of inflammatory cytokines secreted by a spectrum of macrophage phenotypes. The process initially amplifies the pro‐inflammatory response, then accelerates M1‐to‐M2 macrophage polarization transition, yielding a satisfactory pattern of osteo‐immunomodulation during the natural bone healing process. Later, sustained release of Zn^2+^/Sr^2+^ ions with gradual degradation of the 3D scaffold maintains the favorable reparative M2‐dominated immunological microenvironment that supports new bone mineralization. Precise temporal immunoregulation of the bone healing process by the intelligent 3D scaffold enhances bone regeneration in a rat cranial defect model. This strategy paves the way for the application of β‐tricalcium phosphate‐based materials to guide the dynamic inflammatory and bone tissue responses toward a favorable outcome, making clinical treatment more predictable and durable. The findings also demonstrate that near‐infrared irradiation‐derived mild hyperthermia is a promising method of immunomodulation.

## Graphical abstract

The natural bone healing process is modulated by sequential immunological responses, including the pro‐inflammatory initiation stage and later anti‐inflammatory remodeling. Focusing on spatiotemporal osteoimmunomodulation, the study develops a 3D scaffold capable of dynamically coordinating the inflammatory and bone tissue responses by regulating the initiation stage, accelerating the shift from a pro‐ to an anti‐inflammatory, and enhancing the anti‐inflammatory remodeling stage.

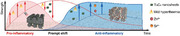

## 1 Introduction

The mainstream strategy for developing artificial bone platforms has stressed the importance of promoting the osteogenic differentiation of the osteoblast lineage.^[^ ^1^ ^]^ As an alternative to autologous and allogeneic bone grafts, β‐tricalcium phosphate (β‐TCP)‐based bioceramic scaffolds have been utilized in orthopedics because of their outstanding biological characteristics, including biocompatibility, osteoconduction, and degradation absorption.^[^ ^2^ ^]^ However, despite these advantages, inconsistent osteogenic effects have often been produced in in vitro and in vivo assays by β‐TCP stimulation, which are partly attributable to the absence of active interaction with the various effector cells involved in the natural bone healing process.^[^ ^3^ ^]^ Recently, there has been increasing recognition that bone tissue repair and regeneration is a complex, metabolically demanding process, heavily associated with the host immune response rather than relying solely on the development of the skeletal system.^[^ ^4^ ^]^ Thus, the use of solely β‐TCP scaffolds has been sidelined. The development of a novel bone substitute biomaterial that can coordinate the multi‐phase, dynamic bone healing process is imperative.

From an osteoimmunological view, inflammatory reactions play a central role in coordinating a biomaterial's interaction with the bone tissue microenvironment, which in turn regulates the subsequent osteogenic activities of cells during the bone regeneration process. Moreover, the inflammation following injury is normally recognized as modular, occurring in three distinct phases that work together to restore the natural bone architecture.^[^ ^5^ ^]^ Bone repair is initiated with the innate immune response, in an early pro‐inflammatory microenvironment, which is essential for defense against pathogens and the recruitment of inflammatory cells such as macrophages with a classically activated inflammatory phenotype (M1).^[^ ^4a^ ^]^ Following this early acute inflammatory stage that occurs within hours to days after implantation, the pro‐inflammatory response starts to fade and macrophages switch to reparative anti‐inflammatory (M2) and angiogenic (M2‐like) phenotypes.^[^ ^6^ ^]^ Such a prompt shift from a pro‐ to an anti‐inflammatory response is vital for the regulation of the second major phase of osteogenic activity.^[^ ^7^ ^]^ Bone remodeling then proceeds until tissue homeostasis is restored, within weeks to months, at which point M2 is the dominant phenotype.^[^ ^4c^ ^]^

While the inflammatory response drives many aspects of tissue regeneration, once it becomes dysregulated, it leads to pathological fibrosis, resulting in the disruption of normal bone tissue architecture.^[^ ^8^ ^]^ It is well known that a prolonged pro‐inflammatory phase can impede bone healing, and current bone biomaterials have been developed to promote the involvement of M2 macrophages while suppressing M1 macrophages.^[^ ^9^ ^]^ However, the immune response is remarkably intricate, and involves a delicate, dynamic balance between heterogeneous M1/M2 populations, pro‐immune/inflammatory processes, and regulatory/suppressive functions.^[^ ^10^ ^]^ The positive influence of pro‐inflammatory cytokines produced during the earliest phase on the maintenance or restoration of bone homeostasis has been increasingly concerned.^[^ ^4a^ ^]^ Very‐low‐levels of pro‐inflammatory cytokines can enhance the ability to prevent or slow down overt inflammation in the very early phase, thus initiating signal transduction to restore homeostasis.^[^ ^11^ ^]^ Pro‐inflammatory cytokines, such as tumor necrosis factor α (TNF‐α), accelerate bone healing when administered promptly at the fracture site after injury and inhibit cartilage formation when administered later.^[^ ^12^ ^]^ After quickly, effectively driving macrophages to switch from the M1 to M2 phenotype, the anti‐inflammatory environment must be maintained in the later phase of bone healing to balance various osteogenic and angiogenic cytokines.^[^ ^4^, ^13^ ^]^ In addition, reactive oxygen species (ROS) are often overproduced in response to pro‐inflammatory mediators, resulting in damage to proteins, DNA, lipids, and lipids membranes, as well as disruption of vital cellular processes, thereby hampering osteogenesis.^[^ ^14^ ^]^ On the other hand, if elevated ROS persist in disturbing homeostasis, the secretion of pro‐inflammatory cytokines is stimulated, causing a delayed switch to the anti‐inflammation program, which may result in severe oxidative stress and an overt inflammatory state, eventually causing the formation of a fibrous capsule that impairs osteogenesis.^[^ ^4a^ ^]^ Therefore β‐TCP–based scaffolds endowed with the capacity to orchestrate multi‐phased inflammatory and bone tissue responses and coordinate ROS scavenging have been developed to promote bone repair and regeneration within a short period.

Febrile temperature in the range of 39 –43 °C can amplify immunomodulatory effects and enhance bone tissue regeneration.^[^ ^15^ ^]^ Moderate hyperthermia may stimulate or suppress cytokine production by macrophages, thereby promoting the early activation of host defenses and preventing prolonged exposure to potentially harmful cytokines.^[^ ^16^ ^]^ Near‐infrared (NIR) irradiation can penetrate tissues to provoke targeted, mild photothermal hyperthermia with spatial and temporal precision.^[^ ^17^ ^]^ Based on this, we developed a 2D Ti_3_C_2_T_x_‐based MXene nanosheet to decorate a 3D‐printed ceramic scaffold. As unique semiconductor materials, Ti_3_C_2_ nanosheets possess desirable NIR photothermal conversion efficiency combined with good biocompatibility.^[^ ^18^ ^]^ Moreover, the intrinsic Ti‐C redox‐active sites of Ti_3_C_2_ exhibit promising capacity for scavenging ROS.^[^ ^19^ ^]^ As a consequence of the catalytic activities toward classical peroxidase substrates, the Ti_3_C_2_ MXene nanosheets may exert enzyme‐like characteristics to catalyze the dismutation reaction of O^2−^• into O_2_ and H_2_O_2_, as well as the decomposition of two molecules of H_2_O_2_ for yielding O_2_ and H_2_O.^[^ ^20^ ^]^ To improve the mechanical and osteogenic properties of the 3D‐printed β‐TCP scaffold, zinc (Zn) and strontium (Sr) ions were co‐incorporated as precursors to partially replace calcium (Ca) ions in a Ca‐deficient apatite composition through aqueous coprecipitation, followed by sintering to prepare co‐substituted ZnSr/β‐TCP (ZST) powders as subsequent 3D‐printed raw materials.^[^ ^21^ ^]^ Meanwhile, the long‐term release of Zn^2+^ and Sr^2+^ ions during the gradual degradation of the scaffold is intended to maintain anti‐inflammatory regulation and stimulate osteoblastgenesis in the later phase of bone regeneration.^[^ ^22^ ^]^

**Scheme 1:**
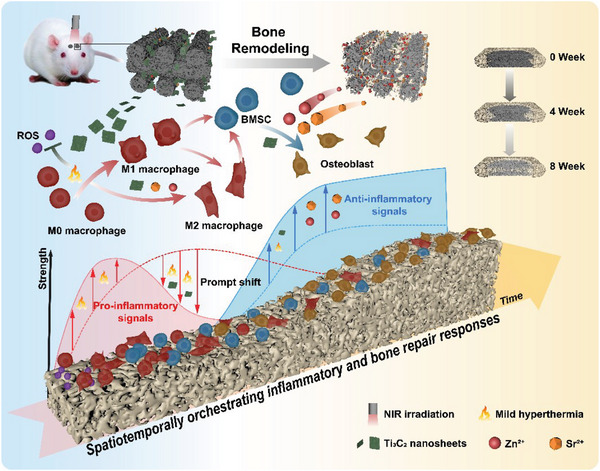
Schematic illustration of Ti_3_C_2_Tx MXene‐decorated 3D‐printed ceramic scaffold for enhancing osteogenesis by spatiotemporally orchestrating inflammatory and bone repair responses.

As illustrated in **Scheme** **1**, NIR irradiation conducted in the early post‐implantation phase is controlled to produce local mild hyperthermia (42 °C ± 1 °C) around the composite scaffold, potentially amplifying host inflammatory mediator expression in parallel with enhanced ROS scavenging by Ti_3_C_2_ nanosheets. Persistent and recurrent NIR irradiation provokes moderate hyperthermia that can further accelerate the M1‐to‐M2 switch of macrophages to create a favorable osteoimmunological microenvironment. Subsequently, both the Ti_3_C_2_ and ceramic‐based scaffold participate in the ensuing bone regeneration with the assistance of mild hyperthermia mediated by periodic NIR irradiation and the gradual release of Ca^2+^, Zn^2+^, Sr^2+^, and PO_4_^3−^ ions beneficial to osteogenesis. In this way, the intelligent 3D ceramic scaffold has the potential to guide the dynamic inflammatory and bone tissue responses toward more favorable outcomes, making clinical treatment more predictable and durable.

## 2 Results

### 2.1 Synthesis and Characterization of the Scaffolds

**Figure 1:**
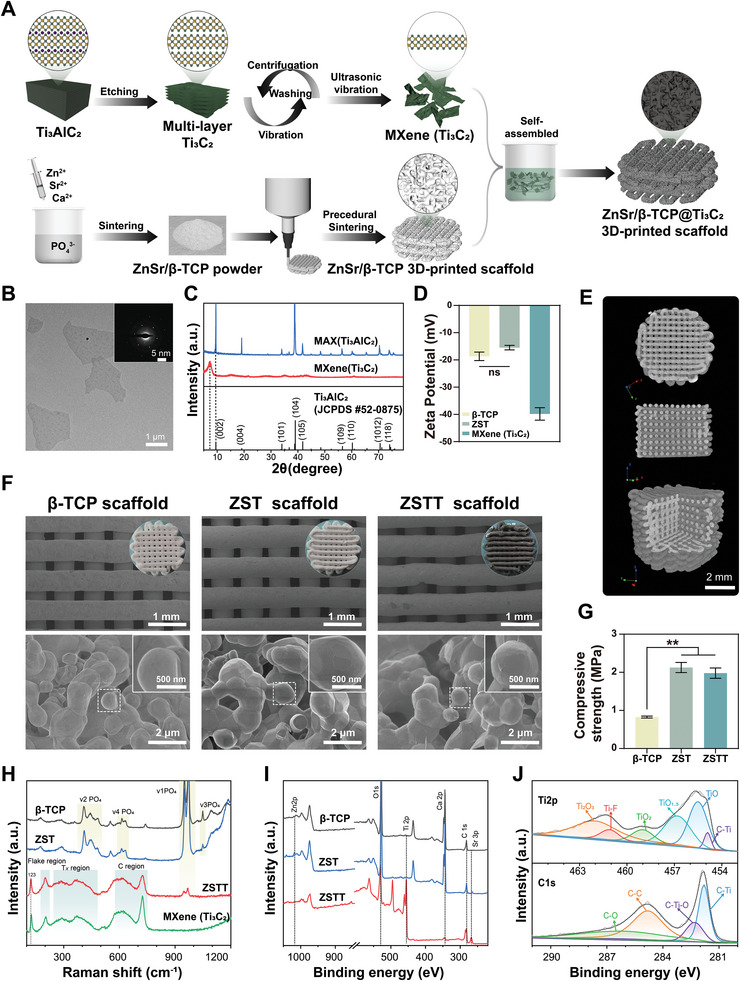
Synthesis and characterization of Ti_3_C_2_‐decorated 3D‐printed scaffolds. A) Schematic illustration of the scaffold fabrication process. B) TEM image and SAED pattern of ultrathin MXene (Ti_3_C_2_) nanosheets. C) XRD patterns of the bulk MAX (Ti_3_AlC_2_) and ultrathin MXene (Ti_3_C_2_) nanosheets. D) Zeta potentials of β‐TCP, ZST, MXene (Ti_3_C_2_), and ZSTT scaffolds. E) Micro‐CT images of scaffolds. F) Digital and SEM images of scaffolds. G) Ultimate compressive strength of the β‐TCP, ZST, and ZSTT scaffolds. H) Raman spectra of MXene (Ti_3_C_2_) nanosheets and scaffolds. I) The full‐scan XPS spectra of scaffolds. J) The narrow‐scan spectra of C1s and Ti2p. The data are presented as the mean ± standard deviation, n = 3. ^**^ *p* < 0.01.

To endow them with NIR‐responsive and ROS scavenging capacities, we decorated 3D‐printed ZnSr/β‐TCP@Ti_3_C_2_ (ZSTT) scaffolds with 2D Ti_3_C_2_ nanosheets, as illustrated in **Figure** **1**A. Sheet‐like Ti_3_C_2_T_x_ multilayers were initially prepared by etching the Al layers from the bulk MAX (Ti_3_AlC_2_) material in LiF/HCl mixed solution for 24 h (Figure S1, Supporting Information). After ultrasonication of the colloidal suspensions, ultrathin Ti_3_C_2_ nanosheets were obtained in a monolayer structure by breaking weak covalent bonds between the layers (Figure 1B); the sheets showed an obvious Tyndall effect with great dispersibility (Figure S2, Supporting Information). The X‐ray photoelectron spectroscopy (XPS) and energy dispersive spectrometer (EDS) mapping results revealed the coexistence of Ti, Al, and C elements in the MAX, while the Al content was substantially decreased after etching (Figures S3 and S4, Supporting Information). The X‐ray diffraction (XRD) patterns showed a distinct shift of the intense (002) peak from the 2θ angle of 9.5° to a lower 7.4° in parallel with the disappearance of the (104) peak of Ti_3_AlC_2_ MAX at the 2θ degree of 39°, indicating the successful fabrication of MXene Ti_3_C_2_ (Figure 1C). Both the transmission electron microscopy images (TEM) and the corresponding selected area electron diffraction (SAED) pattern demonstrated that the prepared 2D Ti_3_C_2_ nanosheets featured a planar topology and hexagonal crystal structure with good crystallinity (Figure 1B). In addition, the zeta potential result of 2D Ti_3_C_2_ nanosheets is −39 ± 2 mV, indicating the excellent stability of Ti_3_C_2_ nanosheets colloidal solutions (Figure 1D).

To prepare the 3D‐printed ink, powders comprising β‐TCP doped with the combined substitution of Zn and Sr ions were fabricated through aqueous coprecipitation followed by heating of the resultant apatite. Scanning electron microscope (SEM) and particle size analysis results showed that the prepared β‐TCP particles were of nano/micro‐scale and their uniformity and agglomeration improved marginally after the coprecipitation of Zn/Sr (Figure S5, Supporting Information). The zeta potential result further indicated there was no significant change in the stability of the powder solution after Zn^2+^/Sr^2+^ doping (Figure 1D). The XRD patterns of the powders matched well with β‐Ca_3_(PO_4_)_2_ (JCPDS #09‐0169) and presented a slight shift to higher scattering angles after co‐substitution of Zn^2+^ and Sr^2+^ for Ca^2+^, indicating an overall reduction of the unit cell lattice parameters compared with those of pure β‐TCP (Figure S6, Supporting Information).^[^ ^23^ ^]^ Then, the ceramic scaffolds were fabricated with designed, precisely managed morphology and an interconnected, macroporous (300–600 µm) structure by 3D printing followed by sintering, as illustrated in various views by micro‐computed tomography (micro‐CT) (Figure 1E). SEM images further indicated the microporous (1–2 µm) structure of the 3D‐printed scaffolds, and no obvious change in microstructure was observed after the addition of Zn and Sr ions. In addition, we observed that the surfaces of the ZSTT crystals were tightly covered with micro/nano‐scale nanosheets after Ti_3_C_2_ deposition through dip coating (Figure 1F). The EDS images of the ZSTT scaffold demonstrated the presence of O, P, Ca, Sr, Zn, Ti, and C elements, indicating that ion‐doping and Ti_3_C_2_ coating were successful (Figure S7, Supporting Information). Further Raman spectrum analysis of the composition indicated the combination of the scaffold and MXene was represented by the distinct Raman features of a resonant peak (≈123 cm^−1^); out‐of‐plane vibrations of Ti, C, and O in the flake region (150–220 cm^−1^); surface group vibrations in the T*_x_* region (230–470 cm^−1^); and carbon vibrations in the region (580–730 cm^−1^) of MXene (Figure 1H).^[^ ^24^ ^]^ The full‐scan XPS spectra showed that Ca, Sr, Zn, P, O, and C elements were found in all of the specimens, whereas Ti was present only in ZSTT due to the MXene coating (Figure 1I). Furthermore, the peak‐fitting results of the narrow‐scan spectra of C1s and Ti2p identified the C‐Ti (281.8 eV), C‐O‐Ti (282.3 eV), C‐C (284.8 eV), and C‐O (286.1 eV) bonds, as well as C‐Ti (454.9 eV), TiO (455.5 eV), TiO_1.5_ (456.8 eV), TiO_2_ (459 eV), Ti‐F (461.1 eV), and Ti_2_O_3_ (462 eV) bonds, in ZSTT specimen (Figure 1J).^[^ ^25^ ^]^ The XRD patterns of all the scaffolds further confirmed the successful substitution of Zn/Sr in β‐TCP and subsequent MXene decorating (Figure S8, Supporting Information). As expected, the compressive strength of the 3D scaffold was significantly increased from 0.86 ± 0.03 MPa to 2.16 ± 0.02 MPa after Zn/Sr doping, and with little loss after further MXene coating (Figure 1G).

### 2.2 Evaluation of the Photothermal Properties, ROS Scavenging Capacity, and Biocompatibility of the Scaffolds

**Figure 2:**
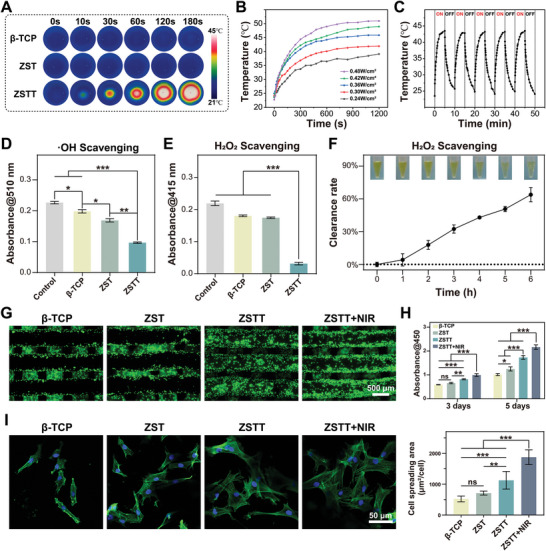
The photothermal properties, ROS scavenging activity, and biocompatibility of the scaffolds. A–C) In culture medium, photothermal images (A) of β‐TCP, ZST, and ZSTT scaffolds, as well as the heating curves (B), and photothermal stability (C) of the ZSTT scaffolds. D,E) ·OH (D) and H_2_O_2_ (E) scavenging activities of scaffolds. F) H_2_O_2_ scavenging curves for the scaffolds. G) Live/dead staining images of the rBMSCs on the scaffolds for 4 days. H) CCK‐8 analysis of rBMSCs on the scaffolds for 3 and 5 days. I) Morphology of rBMSCs on the scaffolds for 4 days. Green channel, F‐actin; blue channel, cell nucleus. The data are presented as the mean ± standard deviation, n = 3. (^**^) *p* < 0.01, (^***^) *p* < 0.001, and ns: no significance.

The photothermal performances of the scaffolds were examined under NIR irradiation with an 808 nm laser at various power intensities, both in dry and wet environments. The thermal images indicated that the temperature of ZSTT could exceed 100 °C within 60 s in a dry state (Figure S9A, Supporting Information) and 45 °C within 180 s when immersed in culture medium (**Figure** **2**A) under continuous exposure to NIR radiation (0.48 W cm^−2^). In comparison, the temperatures of the β‐TCP and ZST scaffolds were unchanged over time, indicating a lack of photothermal ability. The temperature raised in tandem with a gradient increase in power density (0.24–0.48 W cm^−2^) (Figure 2B; Figure S9B, Supporting Information). Moreover, the temperature was elevated to 42 °C ± 1 °C rapidly in wet conditions and 88 °C ± 1 °C in dry conditions under an irradiation density of 0.36 W cm^−2^, which is the maximum laser power threshold for skin tolerance with an exposure time <1000 s.^[^ ^26^ ^]^ Photothermal stability was investigated through cycling experiments; the photothermal curve of ZSTT showed almost no periodic reduction in thermal fatigue resistance during five cycles of radiation exposure (0.36 W cm^−2^), indicating that ZSTT has notable photothermal stability (Figure 2C; Figure S9C, Supporting Information).

The antioxidant ability of the scaffolds was assessed based on their hydroxyl (·OH) and hydrogen peroxide (H_2_O_2_) scavenging effects via spectrophotometry. A dramatic decrease in absorbance ≈510 and 415 nm were detected for the ZSTT scaffolds compared with the other groups, confirming the strong ·OH and H_2_O_2_ scavenging capabilities of the Ti_3_C_2_ nanosheets (Figure 2D,E). In addition, the sustained ROS scavenging potential of the Ti_3_C_2_ nanosheet coating was confirmed by cumulative H_2_O_2_ clearance and visible color fading over time (Figure 2F).

The cytocompatibility of the scaffolds was evaluated in BMSCs using live/dead staining and the CCK‐8 assay. None of the scaffolds caused cell death (Figure 2G) and all increased cell viability upon the extension of culture time (Figure 2H). Specifically, cell viability in the presence of the ZSTT scaffolds with NIR irradiation was significantly better than the viability in the other groups. In addition, cell adhesion after incubation for 4 d on different scaffolds was observed by LSCM and SEM. The assays revealed that BMSCs displayed more pseudopods and a more spread‐out appearance on the ZSTT scaffolds, especially when irradiated with NIR, than the more shrunken cell morphologies observed on the surfaces of the β‐TCP and ZST scaffolds (Figure 2I; Figure S10, Supporting Information).

### 2.3 In Vitro Regulation of the Inflammatory Microenvironment by Various Scaffolds

**Figure 3:**
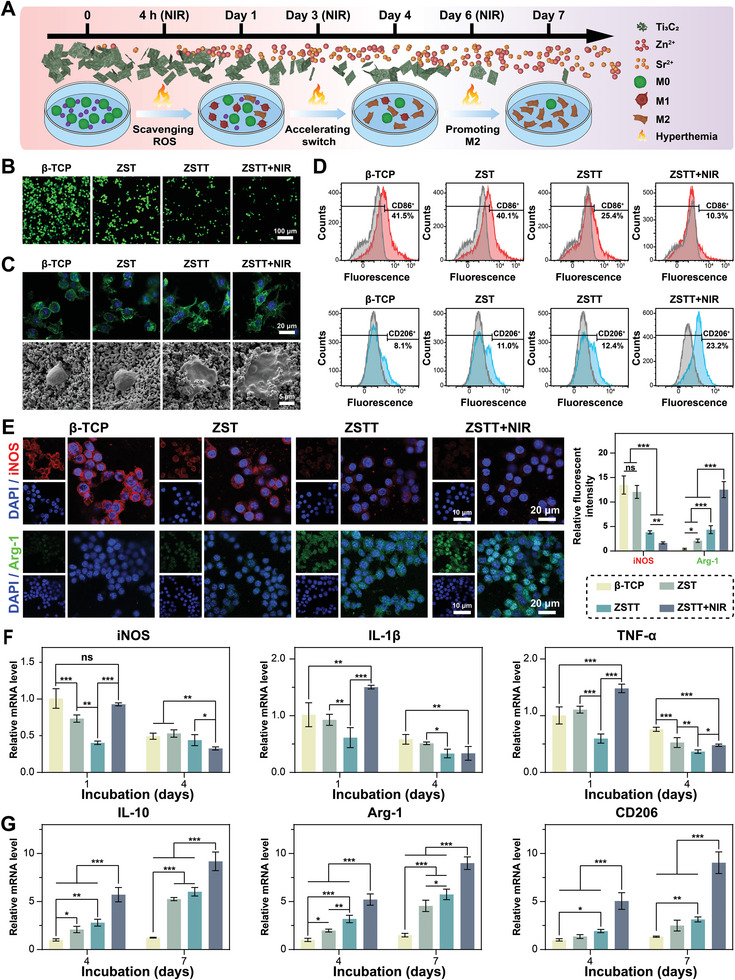
ROS scavenging and immunomodulatory capacity in vitro. A) Schematic illustration of the experimental strategy in vitro. B) The fluorescence images show the intracellular ROS levels of the cultured macrophages. C) The morphology of RAW 264.7 cells cultured on the scaffolds for 4 days. D) Flow cytometric analyses of M1 and M2 polarization markers (CD86 and CD206, respectively). E) Immunofluorescence staining and the corresponding quantification of iNOS (red), Arg‐1 (green), and nuclear (blue) staining of macrophages. F) Gene expression of macrophages cultured for 1, 4, or 7 days. The data are presented as the mean ± standard deviation, n = 3. (^*^) *p* < 0.05, (^**^) *p* < 0.01, and (^***^) *p* < 0.001.

To explore the effects of photothermal stimulation on spatiotemporal immunomodulation, mild hyperthermia (42 °C ± 1 °C) was periodically induced by NIR irradiation (0.36 W cm^−2^) of macrophages seeded on various scaffolds for 10 min per exposure, as illustrated in **Figure** **3**A. The levels of intracellular ROS were reported by the DCFH‐DA probe. The fluorescence intensity of the ZSTT+NIR group was significantly lower than those of the other groups (Figure 3B). The percentages of stained cells in the β‐TCP, ZST, ZSTT, and ZSTT+NIR groups decreased from 89.7% (Lipopolysaccharide‐stimulated) to 76.4%, 67.3%, 58.0%, and 38.1%, respectively, as quantitated by flow cytometry (Figure S11, Supporting Information). SEM and LSCM were applied to observe the morphologies and ultrastructures of macrophages cultured on the scaffolds for 4 days. Cells on the β‐TCP and ZST scaffolds showed an oval shape characteristic of M1 macrophages, whereas those on ZSTT had an M2 morphology, with an elongated spindle‐shape and extended lamellipodia, consistent with previous studies.^[^ ^27^ ^]^ Moreover, the spreading area of the macrophages on the surface of the ZSTT scaffolds was enhanced by NIR irradiation, as was peripheral filopodia formation (Figure 3C).

To evaluate the ability of various scaffolds to modulate the phenotypic transition of macrophages, the phenotypes and activation statuses of macrophages were characterized by flow cytometry, immunofluorescence staining, and q‐PCR. As shown in Figure 3D and Figure S12 (Supporting Information), successively decreasing expression of M1‐associated marker CD86 and increasing expression of M2‐associated marker CD206 were detected in the groups, in the ascending order of β‐TCP, ZST, ZSTT, and ZSTT+NIR. Specifically, the expression of CD86 was reduced by a factor of 4 and CD206 was enhanced threefold in the ZSTT+NIR group compared with the β‐TCP control. A similar trend was also found for the expression of inflammatory cytokines analyzed by immunofluorescence (Figure 3E). It confirmed that there was a significant difference in the expression of iNOS and Arg‐1 with or without NIR irradiation. These results indicated that ZSTT could accelerate the switch from the M1 to the M2 phenotype, especially with the aid of NIR irradiation.

To further clarify the precise control of the scaffolds of the inflammatory process, we analyzed the expression of pro‐inflammatory cytokines (iNOS, IL‐1β, and TNF‐α) (Figure 3F) and anti‐inflammatory cytokines (IL‐10, Arg‐1, and CD206) (Figure 3G) in macrophages at days 1, 4, and 7. On day 1, lower levels of pro‐inflammatory cytokines were expressed in all of the experimental scaffold groups compared with the β‐TCP control group, except for in the ZSTT+NIR group, which exhibited a propensity for M1 polarization with a higher level of pro‐inflammatory cytokines. On day 4, we observed downregulation of the expression of pro‐inflammatory cytokines; we also observed upregulation of anti‐inflammatory cytokines in both the ZSTT and ZSTT+NIR groups compared with in both the β‐TCP and ZST groups, particularly at day 7. These results revealed the potential of ZSTT to induce M2 polarization and higher expression of anti‐inflammatory cytokines compared with β‐TCP and ZST. Furthermore, the mild photothermal stimulation derived from NIR irradiation favored the initial expression of pro‐inflammatory cytokines and an accelerated transition toward the M2 phenotype characterized by higher expression of anti‐inflammatory cytokines after periodic NIR irradiation.

**Figure 4:**
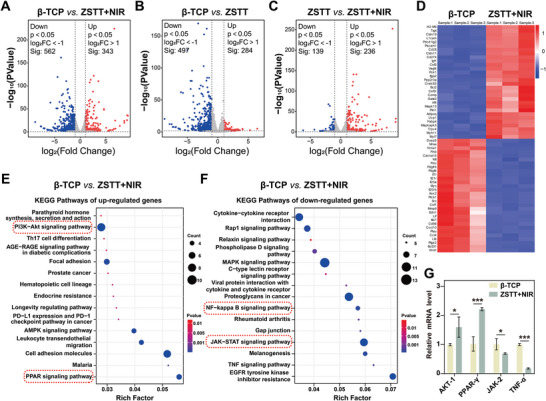
Volcano plot of transcriptomic analysis of differentially expressed genes (DEGs) of MΦs on different samples for 4 days: A) ZSTT with NIR versus β‐TCP, B) ZSTT versus β‐TCP, and C) ZSTT with NIR versus ZSTT. D) Heatmap of DEGs of the 60 significantly upregulated and downregulated genes of MΦs by ZSTT with NIR radiation versus β‐TCP. E,F) Enriched KEGG pathways of macrophages cultured on ZSTT with NIR versus β‐TCP. G) Akt1, PPAR‐γ, JAK‐2, and TNF‐α of MΦs cultured on the ZSTT with NIR versus β‐TCP for 4 days. The data are presented as the mean ± standard deviation, n = 3. (^*^) *p* < 0.05, and (^***^) *p* < 0.001.

To deeply understand the underlying immunomodulation mechanism of the scaffolds with periodic NIR radiation in molecular level, transcriptomic analysis of macrophages cultured on ZSTT scaffolds with and without NIR radiation for 4 days was performed, together with the cells cultured on β‐TCP scaffold as a control group. Volcano plots illustrated the differentially expressed up‐ and down‐regulated genes in each experimental group relative to control at 4‐day (**Figure** **4**A–C), indicating a wide range of gene expression differences of MΦs modulated by the synthetic effects of MXene and photothermal hyperthermia. To focus on the immunomodulatory functions of MXene coating with periodic NIR radiation, the top up‐ and down‐regulated 30 genes of macrophages by ZSTT+NIR versus β‐TCP, ZSTT versus β‐TCP, and ZSTT+NIR versus ZSTT were collected and shown in Figure 4D, Figures S14A and S15A (Supporting Information), respectively. According to the Kyoto Encyclopedia of Genes and Genomes (KEGG) pathway analysis shown in Figure 4E,F, Figures S14B,C and S15B,C (Supporting Information), the signaling pathways of PI3k‐Akt1 and peroxisome proliferator‐activated receptors (PPAR) were up‐regulated, associating with activation of M2 macrophage. Contrarily, the NF−kappa B signaling pathways, and JAK−STAT signaling pathway associated with the M1 phenotype of macrophages were down‐regulated. Furthermore, as shown in Figure 4G, the levels of Akt1 and PPAR‐γ expression were enhanced in ZSTT+NIR, while the expression of JAK‐2 and TNF‐α were down‐regulated. It indicated that the PI3k‐Akt and PPAR signaling pathways were activated by ZSTT+NIR, facilitated M2 polarization. Meanwhile, the NF−kappa B and JAK−STAT signaling pathways were inhibited by ZSTT+NIR, suppressed M1 polarization.

To sum, the in vitro results indicated that the ZSTT scaffolds had effective ROS scavenging capacity and were associated with the upregulation of pro‐inflammatory signals in the initial phase, as well as accelerated M1‐to‐M2 transition, thereby sustaining an anti‐inflammatory environment in subsequent stages.

### 2.4 In Vivo Subcutaneous Immunoregulation in the Early Stage with NIR Irradiation

**Figure 5:**
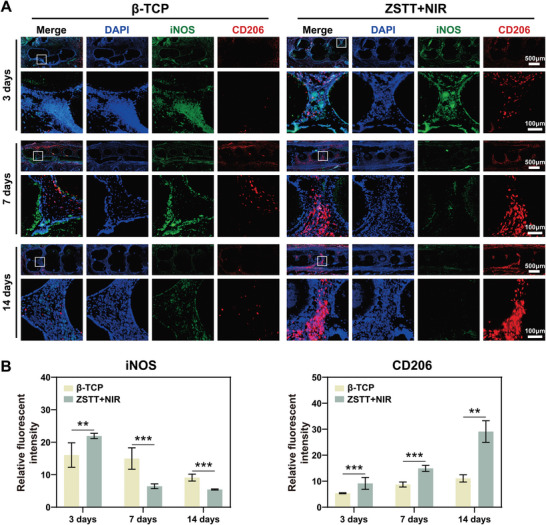
A,B) Immunofluorescence images (A) and the corresponding quantification (B) of iNOS^+^ (stained in green) and CD206^+^ (stained in red) cells in subcutaneous tissues around the scaffolds. The data are presented as the mean ± standard deviation, n = 3. (^**^) *p* < 0.01 and (^***^) *p* < 0.001.

Given the amplifying effect of mild hyperthermia (42 ± 1 °C) on the initial expression of pro‐inflammatory signals in vitro (Figure 3), we performed an in vivo subcutaneous model to further investigate the role of NIR irradiation on early‐stage immunoregulation. Compared with the β‐TCP control, the ZSTT scaffold recruited more inflammatory cells, as shown in the H&E images (Figure S13, Supporting Information) on day 3 after implantation. At the same time, the pro‐inflammatory cytokine iNOS was highly expressed by the cells on the ZSTT scaffold, as indicated by immunofluorescence staining (**Figure** **5**A,B). However, over time, a decrease in inflammatory cell number and iNOS expression was observed along with stronger expression of the anti‐inflammatory marker CD206 in the ZSTT group, indicating a similar effect of NIR‐derived mild hyperthermia on immunoregulation in vivo.

### 2.5 Macrophage‐Mediated Osteogenic Behaviors of rBMSCs

**Figure 6:**
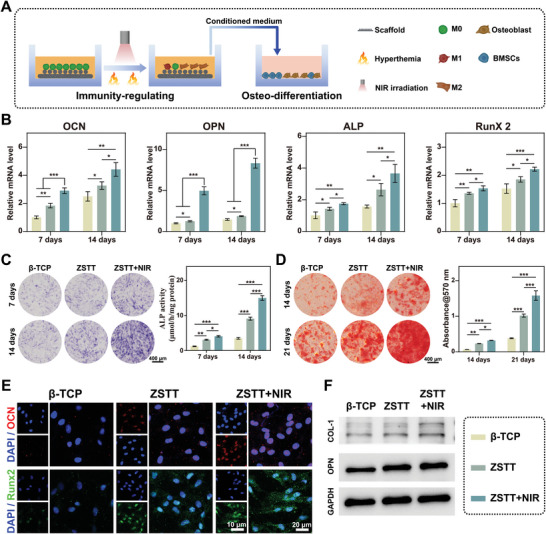
Osteogenic behavior of rBMSCs in vitro. A) Schematic illustration of rBMSCs cultured with conditioned medium collected from the corresponding scaffolds‐treated macrophages. B) Osteogenic gene analysis of rBMSCs, for genes including ALP, Runx2, OCN, and OPN. C) ALP staining at 7 and 14 days. D) Alizarin red staining at 14 and 21 days. E) Immunofluorescence staining for OCN (red), Runx2 (green), and nuclei (blue). F) Western blotting for OPN and COL1. The data are presented as the mean ± standard deviation, n = 3. (^*^) *p *< 0.05, (^**^) *p *< 0.01, (^***^) *p* < 0.001, and ns: no significance.

To reveal the impact of the inflammatory microenvironment on osteogenic differentiation, we cultured rBMSCs with conditioned medium from the corresponding scaffolds‐treated macrophages, as illustrated in **Figure** **6**A. The expression of osteogenic differentiation‐related genes, including alkaline phosphatase (ALP), osteopontin (OPN), osteocalcin (OCN), and runt‐related transcription factor 2 (Runx2) (Figure 6B), together with the ALP activities of the rBMSCs cultured for 7 and 14 days (Figure 6C) were evaluated. ARS staining was used to assay the extracellular matrix mineralization of rBMSCs at days 14 and 21 (Figure 6D). For all the evaluation indicators, the osteogenic differentiation of rBMSCs was enhanced significantly in ZSTT group and further enhanced in ZSTT+NIR group in comparison with β‐TCP group. To further confirm these results, the expression of OPN, Runx2, and COL‐1 was detected on day 7 using immunofluorescence staining (Figure 6E; Figure S16A, Supporting Information) and western blotting (Figure 6F; Figure S16B, Supporting Information), which revealed a similar trend. Taken together, the aforementioned results suggested that macrophages cultured on ZSTT scaffolds with NIR irradiation significantly promoted the osteogenic differentiation and extracellular matrix mineralization of rBMSCs.

### 2.6 Spatiotemporal Modulation of Immuno‐Osteogenesis in a Cranial Defect Repair Model with NIR Irradiation

**Figure 7:**
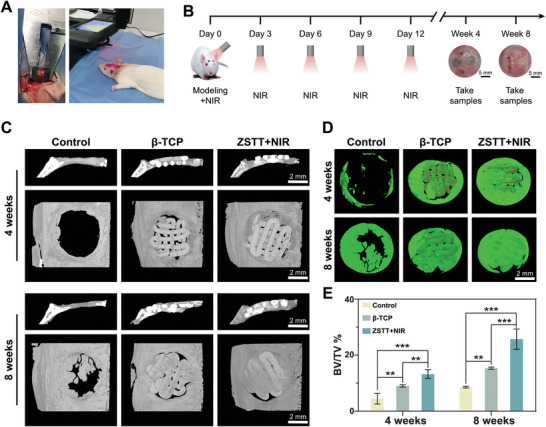
A,B) Schematic diagram (A) of surgery, NIR irradiation, and experimental design (B). C,D) Micro‐CT views (C) of the calvaria and 3D reconstruction images (D). E) Quantitative analysis of BV/TV. The data are presented as the mean ± standard deviation, n = 3. (^**^) *p* < 0.01 and (^***^) *p* < 0.001.

To further assess the in vivo osteogenesis effect associated with spatiotemporal immunoregulation induced by mild hyperthermia, we performed periodic NIR irradiation (at days 0, 3, 6, 9, and 12 after surgery) in a rat cranial defect model during the bone healing process (0–8 weeks), as illustrated in **Figure** **7**A,B. At 4 weeks after implantation, there was almost no notable bone formation in the control group. In contrast, different degrees of bone regeneration were observed in the experimental groups. From the top view, new bone material nearly filled the whole defect in the ZSTT group, although bone mineral density was low. At 8 weeks, cortical bone was observed in the β‐TCP and ZSTT groups, especially in the ZSTT group, with the newly formed confluent bones well bridged and nearly covering the defect area (Figure 7C). The micro‐CT reconstruction technique was applied to reconstruct the 3D image (new bone: green, scaffolds: red, Figure 7D). All bones were reconstructed from the edge toward the center of the defects as the repair process progressed. The β‐TCP group exhibited better osteoinduction properties than the control group. The ZSTT group continued to exhibit the best bone repair efficiency with the most bone tissue inside the scaffold. In addition, a quantitative analysis of bone tissue volume/total tissue volume (BV/TV) was also performed, and the ZSTT scaffold showed the highest percentage of BV/TV compared with the other groups (Figure 7E).

**Figure 8:**
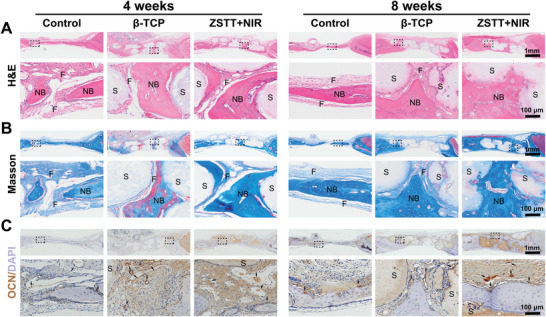
Histological analysis of osteogenesis in vivo. A,B) H&E staining (A) and Masson staining images (B). NB: new bone, F: fibrous tissue, S: scaffold. C) A histological image of OCN staining in the calvarial bone defect tissue.

H&E (**Figure** **8**A), Masson (Figure 8B), and safranin‐O/Fast Green (Figure S13, Supporting Information) staining were used to assess the new bone in the defect area. According to the staining results, the bone defects in the control group were filled with a large amount of fibrotic tissue and a small amount of newly formed bone tissue at 4 weeks. By contrast, more newly formed bone tissue was seen in the β‐TCP and ZSTT groups, although more fibrotic tissue was found in the β‐TCP group. After 8 weeks, the amount of newly formed bone tissue gradually increased in all groups. Regeneration of new bone tissue was fastest in the ZSTT group, and a large amount of bone tissue was generated inside the scaffold at 8 weeks. The fibrotic tissue was almost invisible between the scaffold and the bone tissue in the ZSTT group. Moreover, the expression of OCN was higher in the ZSTT group than in the others (Figure 8C). Finally, H&E histological analysis of the main organs 8 weeks after scaffold implantation indicated no morphological changes or signs of inflammation (Figure S18, Supporting Information).

## 3 Discussion

The natural bone healing process is modulated by sequential immunological responses, including the pro‐inflammatory early initiation stage and later anti‐inflammatory remodeling.^[^ ^13b^ ^]^ The inflammatory responses rely on the release of chemokines or cytokines by a spectrum of macrophage phenotypes.^[^ ^9^ ^]^ Bone fracture or injury impairs the onset of M1 macrophage responses and delays the switch to M2 polarization in response to stimuli, thereby postponing the bone healing process, even resulting in malunion or nonunion.^[^ ^4a^ ^]^ Focusing on immune‐regulated osteogenesis, we attempted to develop an innovative, intelligent bone scaffold capable of dynamically coordinating the inflammatory and bone tissue responses by means of whole‐process modulation, with a focus on the spatiotemporal control of key nodes.

A variety of approaches in biomaterial engineering have been pursued to activate M1 or M2 macrophages to promote osteogenesis, such as physiochemical cue provision by biomaterials, bio‐molecule release, or drug delivery from loaded substrates.^[^ ^28^ ^]^ By contrast, mild hyperthermia can provoke metabolic activity, adhesion, and proliferation of both macrophages and BMSCs, without considering its life‐time and dosage issues.^[^ ^29^ ^]^ In addition, immune activation has been generally identified as an important secondary effect of hyperthermia, which can alter the expression of inflammatory chemokines or cytokines by macrophages by inducing the release of heat shock proteins (Hsps) from cells.^[^ ^17^, ^30^ ^]^ Given that M1 and M2 macrophages can switch phenotypes and that their regulation effects in the bone healing process are time‐dependent, we aimed to guide the transition of macrophages and influence their osteogenic behavior by creating 3D‐printed ceramic scaffolds decorated with Ti_3_C_2_ nanosheets, which are highly sensitive to mild hyperthermia (42 ± 1 °C) induced by NIR irradiation at the local site. The Ti_3_C_2_ nanosheets had excellent ROS scavenging ability, endowing them with the potential to prevent damage to both macrophage polarization and osteoblast function due to oxidative stress caused by elevated ROS levels.^[^ ^19^, ^31^ ^]^

The published results on the effects of hyperthermia on immune responses have been variable and controversial, depending on multiple parameters, including the temperature range (intense hyperthermia of >43 °C versus mild hyperthermia of 39–42 °C) and the manner of induction of hyperthermia (local skin hyperthermia versus whole‐body heating).^[^ ^32^ ^]^ It has been reported that moderate local hyperthermia applied to the skin (39 °C at 2 mm under the skin surface) lasting 3 h could enhance IL‐1 activity with pro‐inflammatory effects via rapid non‐specific activation of immune cells.^[^ ^33^ ^]^ Conversely, exposure of immortalized bone marrow‐derived murine macrophages stimulated with lipopolysaccharide (LPS) to mild heat stress (39 °C for 15 min) rapidly diminished their production of TNF‐α, IL‐6, and IL‐1β by specifically inhibiting the inflammasome.^[^ ^34^ ^]^ In general, studies have shown that mild hyperthermia (39–43 °C) is superior to intense heat (>55 °C) in triggering pro‐inflammatory responses and both necrotic and apoptotic cell death.^[^ ^32c^ ^]^ In the present work, we confirmed that RAW 264.7 cells exhibited sequential phenotype variation with periodic NIR irradiation. The resultant mild hyperthermia (42 ± 1 °C) around the scaffold in this study served as a signal amplifier and even a switch to inflammatory cytokine production. In particular, higher expression of pro‐inflammatory cytokines, such as iNOS, IL‐1β, and TNF‐α driven by M1 macrophages, was promoted by NIR irradiation at the initial stage of cell culture (<24 h) in vitro and in vivo implantation (<3 days). This may be attributable to the Hsp‐mediated macrophage activation mechanism, wherein Hsps, especially Hsp70 and Hsp90, are released into the extracellular microenvironment in response to instantaneous thermal stress to boost the expression of CXCLs, iNOS, and the subsequent pro‐inflammatory interleukins, and to enhance the NF‐κB signaling pathway to increase phagocytotic activity.^[^ ^35^ ^]^ Subsequently, persistent, periodic NIR irradiation (every 3 days for 10 min) remarkably evoked the expression of anti‐inflammatory cytokines (IL‐10, Arg‐1, and CD206), while suppressing pro‐inflammatory signals, indicating the early predominance of M2 macrophages. Day 3 appeared to be critical in the inflammatory modulation timeline, serving as the turning point for reversing the preferential expression of pro‐ or anti‐inflammatory signals. These results were consistent with the results of a previous study that fully polarized M1 and M2 macrophages could reverse their polarization within 3 days.^[^ ^36^ ^]^ However, we recognize that M1 and M2 macrophages are not independent of each other, but exist in a harmonic balance with kinetic transitions over time depending on the interplay between host tissue and bone scaffold local microenvironment. In our study, the PPAR pathway is activated both in the groups of ZSTT+NIR versus β‐TCP and ZSTT versus β‐TCP (Figure 4E; Figure S14B, Supporting Information). It is reported that the activation of PPAR signals can simultaneously suppress JAK‐STAT signals to promote M2 polarization.^[^ ^13b^ ^]^ As shown in Figure 4F and Figure S14C (Supporting Information), the JAK‐STAT signaling pathway was down‐regulated. This indicates that the MXene‐coating may activate PPAR and simultaneously suppress JAK‐STAT signaling pathway. On the other hand, mild hyperthermia promotes M2 polarization through the activation of the PI3k‐Akt signaling pathway.^[^ ^17^ ^]^ In addition, the NF‐kappa B associated with the M1 phenotype of macrophages was down‐regulated in ZSTT+NIR versus ZSTT and ZSTT+NIR versus β‐TCP.^[^ ^37^ ^]^ Thus, NIR irradiation could further enhance the anti‐inflammatory effect of MXene‐coating. In this context, the mild hyperthermia generated through NIR irradiation could be a promising way to modulate inflammation with site‐specific control at a requisite time point, thereby programing the bone healing process.

The results of both the in vitro macrophage/BMSC cultures and in vivo cranial grafts confirmed that the best osteogenic effects were obtained with the spatiotemporal and sequential immunomodulation mediated by ZSTT with periodic NIR irradiation. Our results further demonstrated the indispensable role of chronological amplification of pro‐inflammatory signals followed by accelerated M1‐to‐M2 transition for eventual bone formation and the restoration of tissue homeostasis. M1 macrophages contribute to osteogenesis through the secretion of cytokines such as C‐C chemokine ligand 2, CXC motif ligand 8, and stromal cell‐derived factor 1, which recruit mesenchymal stem cells, osteoprogenitors, and vascular progenitor cells.^[^ ^38^ ^]^ M2 macrophages secrete anti‐inflammatory cytokines that drive the expression of bone morphogenetic proteins, vascular endothelial growth factors, and transforming growth factor β to enhance the osteogenic differentiation of mesenchymal stem cells.^[^ ^38a^ ^]^ Nevertheless, inappropriately early or late activation of M2 macrophages can impair wound healing.^[^ ^39^ ^]^ Superior vascularization compared with controls in a subcutaneous implantation model could be realized by the release of M1‐stimulating interferon γ‐gamma (IFNγ) compared with controls in a subcutaneous implantation model, whereas no effect was observed with the dual delivery of M2‐promoting IL‐4, probably due to the simultaneous rather than consecutive activation of M1 and M2 macrophages.^[^ ^36^ ^]^ Similar research has been conducted in a previous study, regarding the sequential delivery of IFNγ followed by simvastatin by a calcium phosphate coating.^[^ ^40^ ^]^ We suggest that, compared with drugs or chemokines, the mild hyperthermia derived from NIR irradiation is a better stimulus for the M1/M2 switch, potentially avoiding M1/M2 overlap and inappropriate activation. In addition, sustained release of Zn^2+^ and Sr^2+^ ions with the gradual degradation of the 3D scaffold maintained pro‐reparative stimuli at the defect site in the following weeks, facilitating bone formation and remodeling until the restoration of bone homeostasis.

## 4 Conclusion

MXene Ti_3_C_2_ nanosheets were coated on the surfaces of 3D‐printed ceramic scaffolds, comprising a porous β‐TCP based biomaterial with partial Zn^2+^/Sr^2+^ ion‐substitution of Ca^2+^. With its excellent ROS scavenging based on the enzyme‐mimicking property and NIR responsivity through Ti_3_C_2_ decorating, the scaffold exhibited dynamic osteoimmune‐regulating ability. Especially, the expression of pro‐inflammatory cytokines was amplified by initial NIR irradiation in the early phase, followed by acceleration of the transition from the M1 to M2 phenotype of macrophages with the assistance of synthetic effect of MXene coating and mild hyperthermia possibly by the activation of the PI3k‐Akt and PPAR signaling pathways. By orchestrating the inflammatory response with the intelligent 3D scaffold over the course of natural bone healing, we promoted an optimal bone regeneration process in a rat cranial defect repair model with a short duration. Our findings suggest that periodic NIR irradiation‐induced mild hyperthermia (42 ± 1 °C) could be a promising approach to spatiotemporally modulate inflammation. This work paves the way for the application of β‐TCP–based materials in bone grafting to achieve well‐orchestrated inflammatory and bone repair responses.

## 5 Experimental Section

### Synthesis of Ti_3_C_2_‐Decorated 3D‐Printed Ceramic Scaffolds

Unless specifically stated, all of the reagents were bought from Sinopharm Chemical Reagent (Shanghai, China). Suspensions of 2D Ti_3_C_2_ nanosheets were prepared according to the minimally intensive layer delamination method with some modifications. Briefly, Ti_3_AlC_2_ (11 Technology, China) powder was slowly added into a premixed aqueous solution of LiF (Macklin, China) and 9 m HCl (Macklin, China) solution, and stirred at 350 rpm for 24 h at 35 °C. The resulting suspension was washed with deionized water, shaken with a vortex mixer, and centrifuged several times until the pH exceeded 6. Then, the sediment was redispersed in deionized water and bath‐sonicated for 1 h to facilitate exfoliation. The concentration of the suspension, obtained by measuring the weight of the Ti_3_C_2_ powder after vacuum freeze‐drying, was ≈10 mg mL^−1^.

Pure β‐TCP and ZST powders were prepared via a coprecipitation method. The molar ratio of Ca‐P or (Ca + Zn + Sr)/P was 1.5:1, and the molar ratio of Zn‐Sr‐Ca was 0.025:0.025:0.95. To achieve this, CaCl_2_ (31.63 g), SrCl_2_⋅6H_2_O (2.00 g), and ZnCl_2_ (1.02 g) were added to 150 mL of ultra‐pure water to prepare a Zn‐Sr‐Ca solution. (NH_4_)_2_HPO_4_ (26.41 g) was added to 100 mL of ultra‐pure water to obtain a phosphate solution. Then, the Zn‐Sr‐Ca solution was added to the phosphate solution, dropwise. The solution pH was adjusted to 7.0 with NH_3_H_2_O. After 2 h of stirring, the solution was kept at room temperature for 24 h to continue the reaction. The resulting sediment was washed with deionized water and dried at 80 °C in a drying oven. Finally, the powder was sintered at 800 °C for 3 h.

All 3D‐printed scaffolds were fabricated by 3D Discovery (Regenhu, Switzerland). Powder (3 g) and 5% polyvinyl alcohol solution (3 g) were mixed to produce ink, which was loaded into a 3D printer tube with a V‐shaped nozzle (inner diameter of 0.5 mm). Then, a pre‐designed file was imported into 3D Discovery, and the scaffolds were printed. After the 3D‐printed scaffolds were sintered (1000 °C, 3 h), the scaffolds were soaked in Ti_3_C_2_ aqueous solution at 1.0 mg mL^−1^ for 48 h and dried for 12 h in a vacuum to obtain ZSTT scaffolds.

### Characterization of Ti_3_C_2_ Nanosheets, Ceramic Powders, and Scaffolds

To generate SEM images and perform EDS and element mapping, the study used a scanning electron microscope (Regulus8100, Japan). TEM images were observed with a transmission electron microscope (TalosF200i, USA). Zeta potential analysis was conducted with a Zetasizer Nano ZS90 (Malvern, UK). The size distribution analysis was performed with a Winner 2000ZDE (Jinan Winner Particle Instrument Stock, China). XPS was recorded on an X‐ray photoelectron spectrometer (AXIS SUPRA+, Japan). XRD analysis was performed on an X'Pert diffractometer (PANalytical, Netherlands). Raman spectra were recorded on a Raman microscope (InVia‐Reflex, Britain) with the 785 nm line. A NIR laser was produced using an 808 nm high‐power multimode pump laser (Xi'an Hexuer Rade Laser Technology, China). Temperature detection and thermal‐image recording were conducted on an infrared thermal imaging instrument (HM‐TP42‐3AQF/W, China).

The ·OH scavenging capacity of the scaffold was measured with a mixture of 1 mL salicylic acid‐ethanol solution (10 mm), 1 mL FeSO_4_•7H_2_O‐ethanol solution (10 mM), and 0.3 mL H_2_O_2_ at a concentration of 30%. The scaffold was immersed in the mixture for 5 h at 37 °C, and then 1 mL of the supernatant was pipetted into a microplate to measure its absorbance at 510 nm using a microplate reader. H_2_O_2_ assay kits (Beyotime, China) were used to test total anti‐oxidation ability.

To examine the ROS scavenging capacity of the scaffold, LPS was added to a macrophage culture medium. The macrophages were incubated with 2′,7′‐dichlorodihydrofluorescein diacetate (DCFH‐DA) for 30 min after coculturing with scaffolds for 1 day. The stained cells were observed under an inverted fluorescence microscope (SOPTOP, China).

### Cell Culture and Biocompatibility

Mouse macrophages (RAW 264.7) and rat bone marrow‐derived mesenchymal stem cells (rBMSCs) were purchased from Cyagen Biosciences (Guangzhou). In a controlled incubator at 37 °C with an atmosphere of 5% CO_2_, both the rBMSCs and RAW 264.7 cells were cultured in Dulbecco's modified Eagle's medium (DMEM, Gibco, USA) containing 10% fetal bovine serum (FBS, Gibco, USA) and 1% antibiotics (HyClone, USA). To study macrophage polarization, RAW 264.7 cells were seeded in the wells of a 24‐well plate in which β‐TCP, ZST, and ZSTT scaffolds had been placed (n = 3 in each group), with a seeding density of 5 × 10^4^ cells well^−1^. NIR irradiation was performed on days 0, 3, 6, 9, and 12 after seeding. To reveal the impact of the inflammatory microenvironment on the osteogenic differentiation of rBMSCs, the conditioned medium from macrophages at 1, 4, and 7 days was collected and prepared for BMSC‐conditioned culture.

The CCK‐8 method was used to analyze proliferation and cytotoxicity after culturing on scaffolds for 3 and 5 days. The rBMSCs were seeded on the scaffolds for 48 h, and a live/dead cell staining kit (Yeasen, China) was used for cell staining. After 30 min, the stained cells were observed under an inverted fluorescence microscope (SOPTOP, China). On day 4, the cell‐loaded scaffolds were fixed with 4% paraformaldehyde and incubated with fluorescein isothiocyanate (FITC)‐phalloidin for 45 min, then with DAPI for 10 min, and finally observed using a laser scanning confocal microscope (LSCM, Celldiscoverer 7, Germany). After 4 days of incubation, the cell‐loaded scaffolds were fixed with 4% glutaraldehyde for 1 h at 4 °C. The samples were then dehydrated in an ethanol gradient, followed by vacuum drying. After coating with gold, the samples were observed using SEM to determine the morphologies of the adhered cells.

### Immunofluorescence Staining

The expression of inflammatory markers in macrophages and the expression of osteogenic signals in rBMSCs were evaluated by immunofluorescence staining. Briefly, the cells were fixed with 4% paraformaldehyde for 15 min, followed by permeabilization with 0.2% Triton X‐100 for 15 min and incubation with blocking solution (PBS supplemented with 1% bovine serum albumin, 0.1% Tween 20, and 0.3 m glycine) for 1 h. Subsequently, the primary antibodies against CD206 (Abcam), iNOS (Abcam), OCN (Abcam), and OPN (Abcam) were added to the scaffolds, which were incubated at 4 °C overnight. After rinsing with PBS, the FITC‐conjugated or tetramethylrhodamine isothiocyanate‐conjugated secondary antibody was added and incubated at 37 °C for 1 h, followed by 10 min of nuclear staining with DAPI. The samples were observed and photographed with a Celldiscoverer 7.

### Gene expression Analysis

Total cellular RNA was isolated using the Universal RNA Extraction Kit (AG21011, China) and reverse‐transcribed into complementary DNA using the Evo M‐MLV RT Mix Kit (AG11728, China). Real‐time PCR was carried out using gene‐specific primers and SYBR Green (AG11701, China) on a LightCycler 480 (Roche, Switzerland). The housekeeping gene glyceraldehyde‐3‐phosphate dehydro‐genase (GAPDH) was used as an endogenous reference gene to normalize the calculation with the 2^−ΔΔCt^ method. The primer sequences (Tsingke Biotech, China) are listed in Table S1 (Supporting Information).

### Flow Cytometry

To examine the ROS scavenging capacity of the scaffold, the macrophage culture medium was treated with LPS. The macrophages were incubated with DCFH‐DA for 30 min after culturing on the scaffolds for 1 day. Flow cytometric analysis was performed with a flow cytometer (Cytek Aurora, USA) to evaluate the percentage of DCFH‐DA^+^ and DCFH‐DA^−^ cells on the scaffold.

After 4 days’ culture on the scaffolds, the macrophages were isolated by trypsinization and then incubated with PE‐conjugated anti‐CD206 (Abcam) and APC‐conjugated anti‐CD86 (Abcam) for 1 h. Finally, the expression of the inflammatory markers was detected with the Cytek Aurora.

FlowJo 10.8.1 software (Tree Star) was used to analyze the FCS file output from the Cytek Aurora flow cytometer.

### Transcriptome Sequencing and Data Analysis

RAW 264.7 cells were seeded in the wells of a 24‐well plate in which β‐TCP and ZSTT scaffolds had been placed (n = 3 in each group). NIR irradiation was performed at 4 h and 3days. RAW 264.7 cells were treated with TRIzol reagent (Beyotime Biotechnology) and stored at −80 °C before sequencing. The RNA sequencing was performed using an Illumina HiSeq X10 (Illumina, USA). The gene expression value was transformed as log 10 [TPM (transcripts per million reads) +1]. The RNA sequencing data were normalized via the fragments per kilobase per million reads method.

### Evaluation of the Osteogenic Differentiation of rBMSCs

After 7 and 14 days of culture, the ALP activity of rBMSCs was measured using an ALP kit (Beyotime, China). Briefly, cells were fixed with 2.5% glutaraldehyde for 8 min, washed with PBS, and incubated with ALP staining solution (Beyotime, China) at room temperature for 20 min. Finally, the staining was stopped by washing three times in PBS. After 14 and 21 days of culture, alizarin red staining was performed to detect the formation of calcium nodules. The rBMSCs were washed twice with PBS and fixed with 4% paraformaldehyde for 20 min, then stained with alizarin red staining solution. The calcium nodules were dissolved with perchloric acid and quantified at 570 nm using the microplate reader (Tecan Spark, USA).

Total proteins from rBMSCs were extracted via radioimmunoprecipitation assay lysis, and the protein concentrations were determined by bicinchoninic acid assay. Equal amounts of proteins were subjected to sodium dodecyl sulfate‐polyacrylamide gel electrophoresis and transferred onto a pure nitrocellulose blotting membrane. Membranes were blocked with 5% (w v^−1^) skimmed milk at room temperature for 1 h, and then incubated with primary antibodies for COL‐1 (Abcam) and OPN (Abcam) overnight at 4 °C. The membranes were marked with appropriate peroxidase‐conjugated secondary antibodies (Beyotime, China). Molecular Image ChemiDoc MP system (Bio‐Rad, USA) was used to detect the signal.

### Animal Grouping and Surgery

All animal procedures were approved by the Animal Ethical Committee of Shandong Provincial Hospital Affiliated to Shandong First Medical University (No. 2023–033), and performed according to the National Institutes of Health Guide for the Care and Use of Laboratory Animals (1996). Animal surgery was carried out on male Sprague Dawley rats (10 weeks old, weighing 290 g ± 15 g).

A subcutaneous scaffold implantation model was established in rats to evaluate the effect of regulating the early inflammatory microenvironment. In brief, 9 male Sprague Dawley rats were anesthetized using 2.0% sodium avertin/distilled water (dose of 300 mg kg^−1^). The middle of the back was shaved and disinfected with entoiodine, and two symmetrical subcutaneous pockets were created by making vertical incisions (1.5 cm long). The β‐TCP and ZSTT scaffolds were inserted, and the wounds were sutured and closed. The day of surgery was considered day 0. NIR (808 nm, 0.36 W cm^−2^) irradiation was administered for 10 min on days 0, 3, 6, 9, and 12. All rats were sacrificed at 3, 7, or 14 days.

To investigate the effect of scaffold‐guided bone regeneration, the Sprague Dawley rats were randomly divided into three groups (Control, β‐TCP, and ZSTT+NIR) and anesthetized by intraperitoneal injection of 2.0% sodium avertin/distilled water (dose of 300 mg kg^−1^). After fully exposing the skull, the study ground away 5 mm of bone tissue on both sides of the sagittal suture of the skull using an electric drill. Then, the incision was closed by suturing the periosteum and skin separately. Finally, in vivo photothermal therapy was performed on days 0, 3, 6, 9, and 12. All rats were sacrificed at 4‐ or 8‐weeks post‐procedure.

### Micro‐CT Analysis

All samples were scanned using a micro‐computed tomography (CT) system (Siemens, Germany) to analyze new bone formation at the site of the calvarial bone defects. The undecalcified samples were examined at a resolution of 10 µm. The micro‐CT software (Siemens, Germany) was used for 3D reconstruction. Then, the relative bone volume/tissue volume (BV/TV) in the defect regions was used to calculate new bone formation using the auxiliary software of the Siemens micro‐CT system.

### Histological Analysis

Serial 5 µm‐thick cross‐sections of the subcutaneous tissue samples were subjected to hematoxylin and eosin (H&E) and immunofluorescence staining. The H&E staining was performed according to the manufacturer's protocol (Servicebio, China). For immunofluorescence staining, homologous double immunofluorescence was performed. Paraffin sections were deparaffinized, subjected to antigen recovery using one‐step dewaxing/antigen retrieval buffer, and incubated with specific primary antibodies: anti‐iNOS (Abcam) and anti‐CD206 (Abcam), followed by corresponding secondary antibodies and DAPI. The antibodies were incubated and eluted in sequence.

To evaluate in vivo osteogenesis, calvarial tissue samples were collected at 4 and 8 weeks. Serial 5 µm‐thick cross‐sections of the samples were subjected to H&E, Masson, safranin‐O/Fast Green, immunohistochemical, and immunofluorescence staining. The H&E, Masson, and safranin‐O/Fast Green staining procedures were performed according to the manufacturers’ protocols. In addition, immunohistochemical staining was performed with OCN (Abcam) primary antibodies.

### Statistical Analysis

Every experiment was replicated independently three times, and the results were represented as the mean ± standard deviation. Variations between multiple groups were assessed using one‐way analysis of variance (ANOVA). P values were calculated using GraphPad Prism 6 software. Major differences were indicated as (^*^) *p* < 0.05, (^**^) *p* < 0.01, and (^***^) *p* < 0.001; ns denotes no statistical significance.

## Conflict of Interest

The authors declare no conflict of interest.

## Supporting information

Supporting information

## Data Availability

The data that support the findings of this study are available from the corresponding author upon reasonable request.
