# Supplementary material for: Ti3C2Tx MXene‐Decorated 3D‐Printed Ceramic Scaffolds for Enhancing Osteogenesis by Spatiotemporally Orchestrating Inflammatory and Bone Repair Responses
Source: Adv Sci (Weinh). 2024 Jul 8;11(34):2400229. doi: 10.1002/advs.202400229 (PMC11425883; doi:10.1002/advs.202400229)
Supplement: Supplementary file 1 — Supporting information [file ADVS-11-2400229-s001.docx]

**Ti_3_C_2_T_x_ MXene-decorated** **3D-printed Ceramic Scaffolds for Enhancing Osteogenesis by** **Spatiotemporally Orchestrating Inflammatory and** **Bone Repair Responses**

*Benzhao Huang,* *Shishuo Li, Shimin Dai, Xiaoqing Lu, Peng Wang, Xiao Li, Zhibo Zhao, Qian Wang, Ningbo Li, Jie Wen, Yifang Liu, Xin Wang,* *Zhentao Man ^*^, Wei Li ^*^, Bing Liu ^*^*

B. Huang, N. Li, J. Wen, Y. Liu, X. Wang, B. Liu

Department of Stomatology, Shandong Provincial Hospital Affiliated to Shandong First Medical University, Jinan, Shandong, 250021, China

E-mail: liubing@sdfmu.edu.cn

B. Huang, S. Li, S. Dai, X. Lu, P. Wang, X. Li, Z. Zhao, Z. Man, Wei Li

Department of Joint Surgery, Shandong Provincial Hospital Affiliated to Shandong First Medical University, Jinan, Shandong, 250021, China

E-mail: greatli2000@163.com; mztqd1984@163.com

B. Huang, N. Li, J. Wen, Y. Liu, X. Wang, B. Liu

Medical Science and Technology Innovation Center, Shandong First Medical University & Shandong Academy of Medical Sciences, Jinan, Shandong, 250117, China

N. Li, J. Wen, Y. Liu, X. Wang, B. Liu

School of Stomatology, Shandong First Medical University & Shandong Academy of Medical Sciences, Jinan, Shandong, 250021, China

X. Lu, Z. Man, Wei Li

College of Sports Medicine and Rehabilitation, Shandong First Medical University & Shandong Academy of Medical Sciences, Jinan, Shandong, 250117, China

Z. Man

Endocrine and Metabolic Diseases Hospital of Shandong First Medical University, Shandong Institute of Endocrine and Metabolic Diseases, Jinan, Shandong Province 250062, China

Q. Wang

College of Engineering and Applied Sciences, National Laboratory of Solid State Microstructure, and Collaborative Innovation Center of Advanced Microstructures, Nanjing University, Nanjing 210023, P. R. China

**Keywords:**

3D-printed ceramic scaffolds, MXene decoration, zinc/strontium ion-substitution, near-infrared responsivity, mild hyperthermia, spatiotemporal regulation of inflammatory, bone regeneration


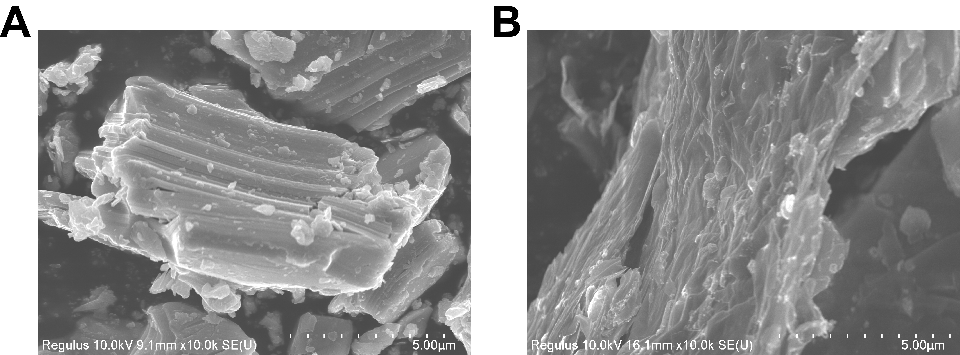


**Figure S1.** SEM images of bulk Ti_3_AlC_2_ ceramic (A) and multilayer Ti_3_C_2_ (B).


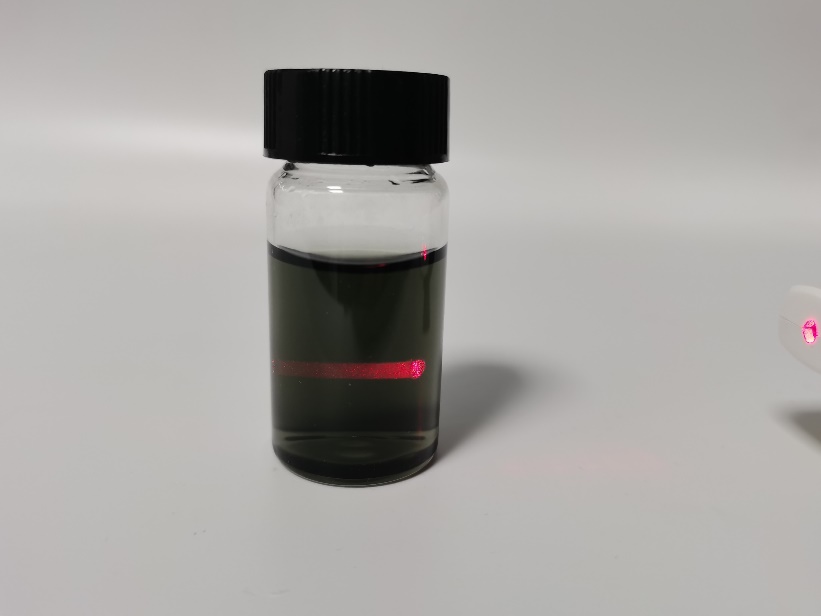


**Figure S2.** Tyndall effect of the 2D MXene (Ti_3_C_2_) nanosheets solution.


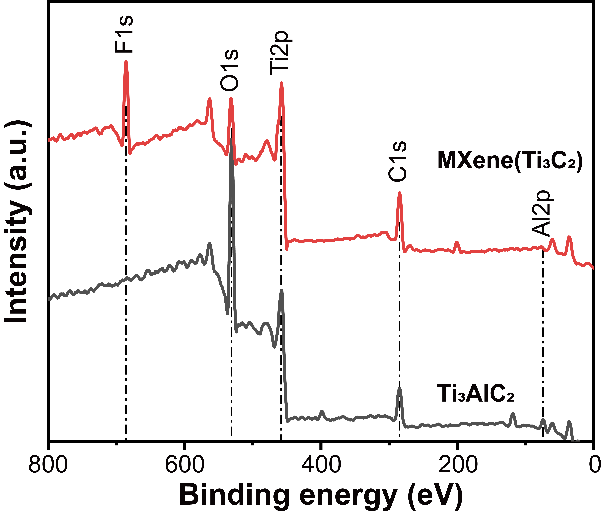


**Figure S3.** The full-scan XPS spectra of MXene (Ti_3_C_2_) and Ti_3_AlC_2_.


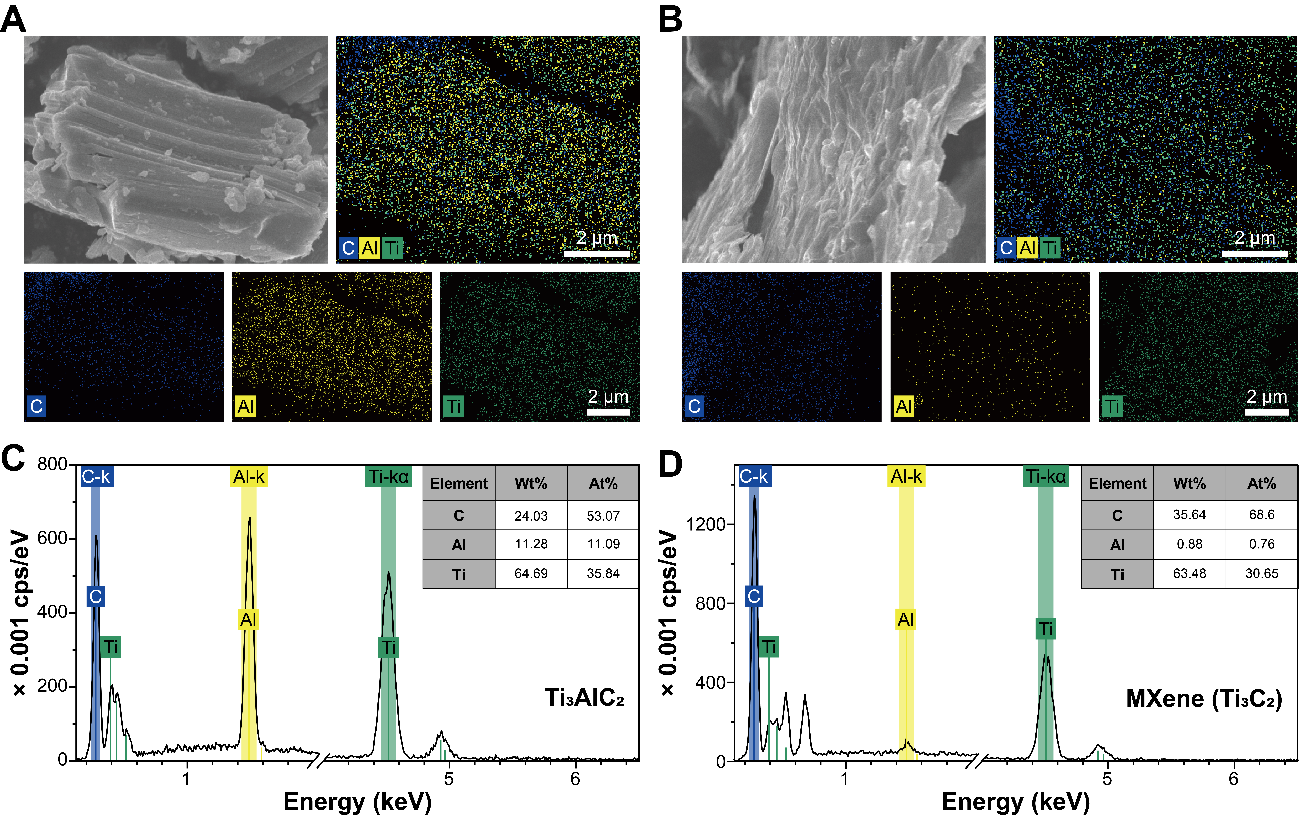


**Figure S4.** Elemental mapping images and EDS profiles of Ti_3_AlC_2_ (A and C) and MXene (Ti_3_C_2_) (B and D).


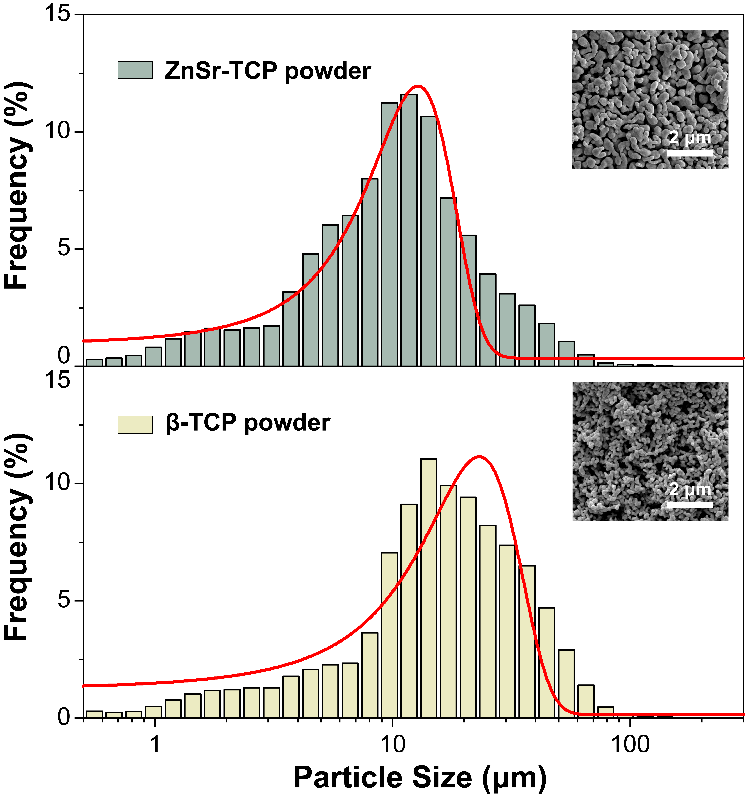


**Figure S5.** SEM and particle size distribution of β-TCP and ZnSr-TCP powder.


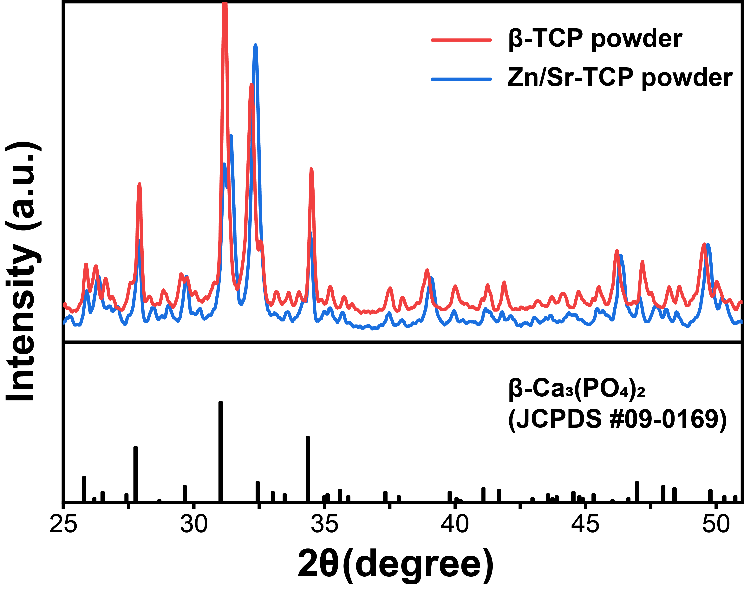


**Figure S6.** XRD patterns of β-TCP and ZnSr-TCP powder.


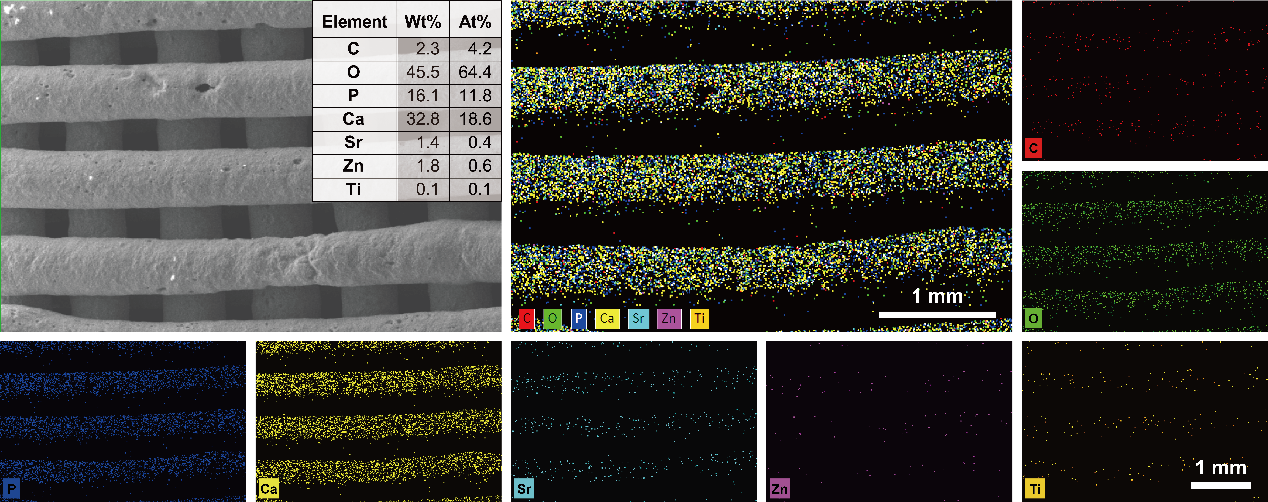


**Figure S7.** Elemental mapping images of ZSTT scaffold.


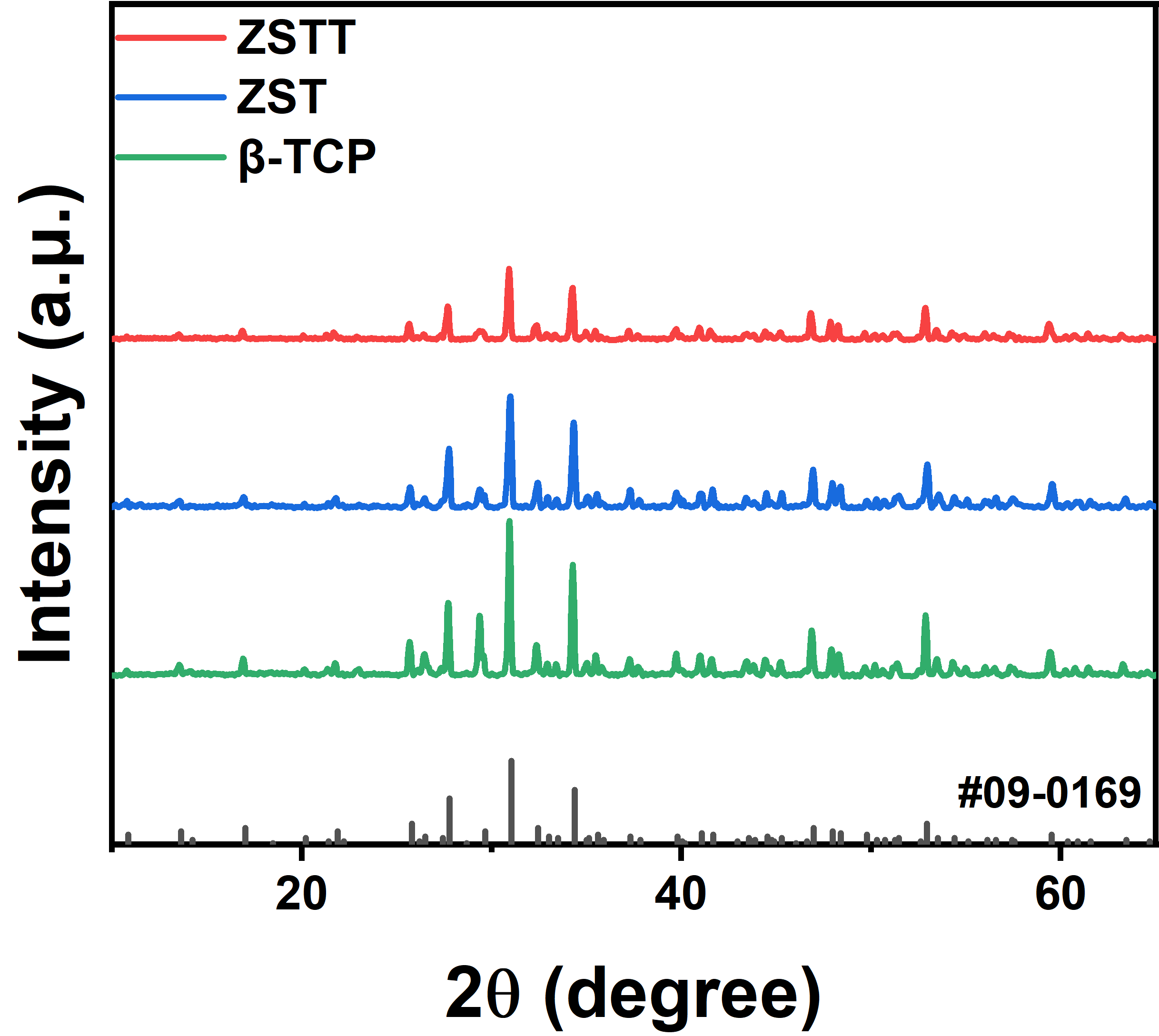


**Figure S8.** XRD patterns of β-TCP, ZST, and ZSTT scaffolds.


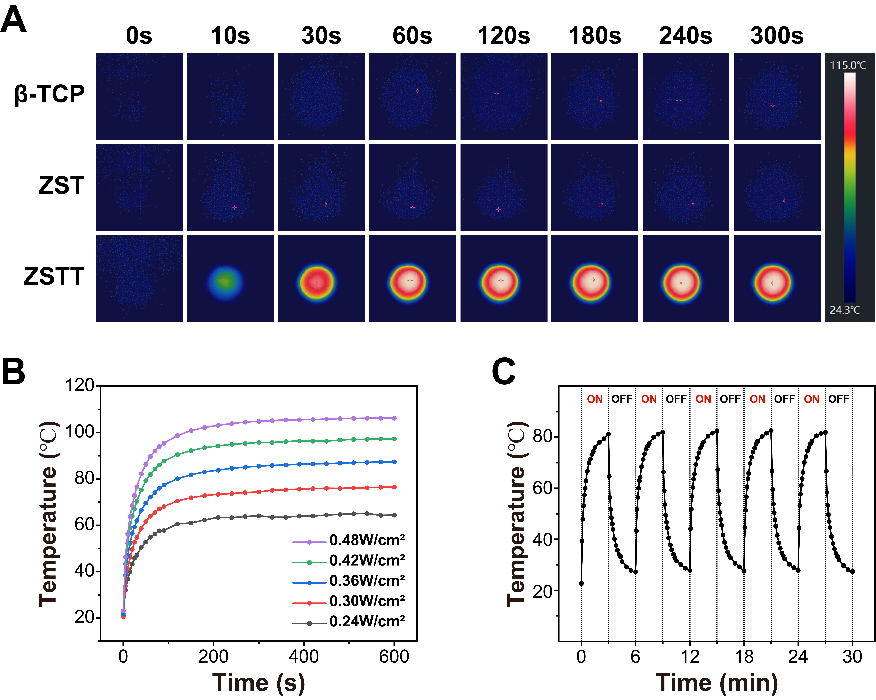


**Figure S9.** A) Photothermal images of β-TCP, ZST, and ZSTT scaffolds. B) Heating curves of ZSTT scaffolds at different radiation powers. C) Photostability of ZSTT scaffolds exposed to an 808 nm laser during five laser on/off cycles.


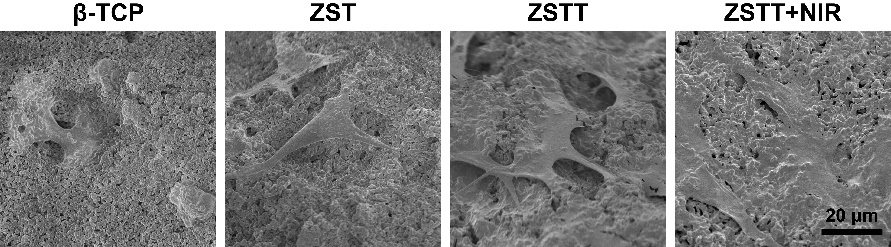


**Figure S10.** SEM images of the BMSCs cultured on scaffolds for 4 days.


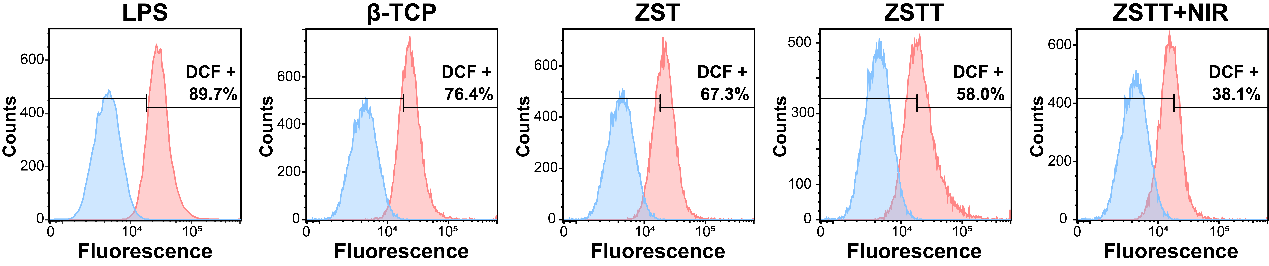


**Figure S11.** Flow cytometric analyses of macrophage intracellular ROS levels.


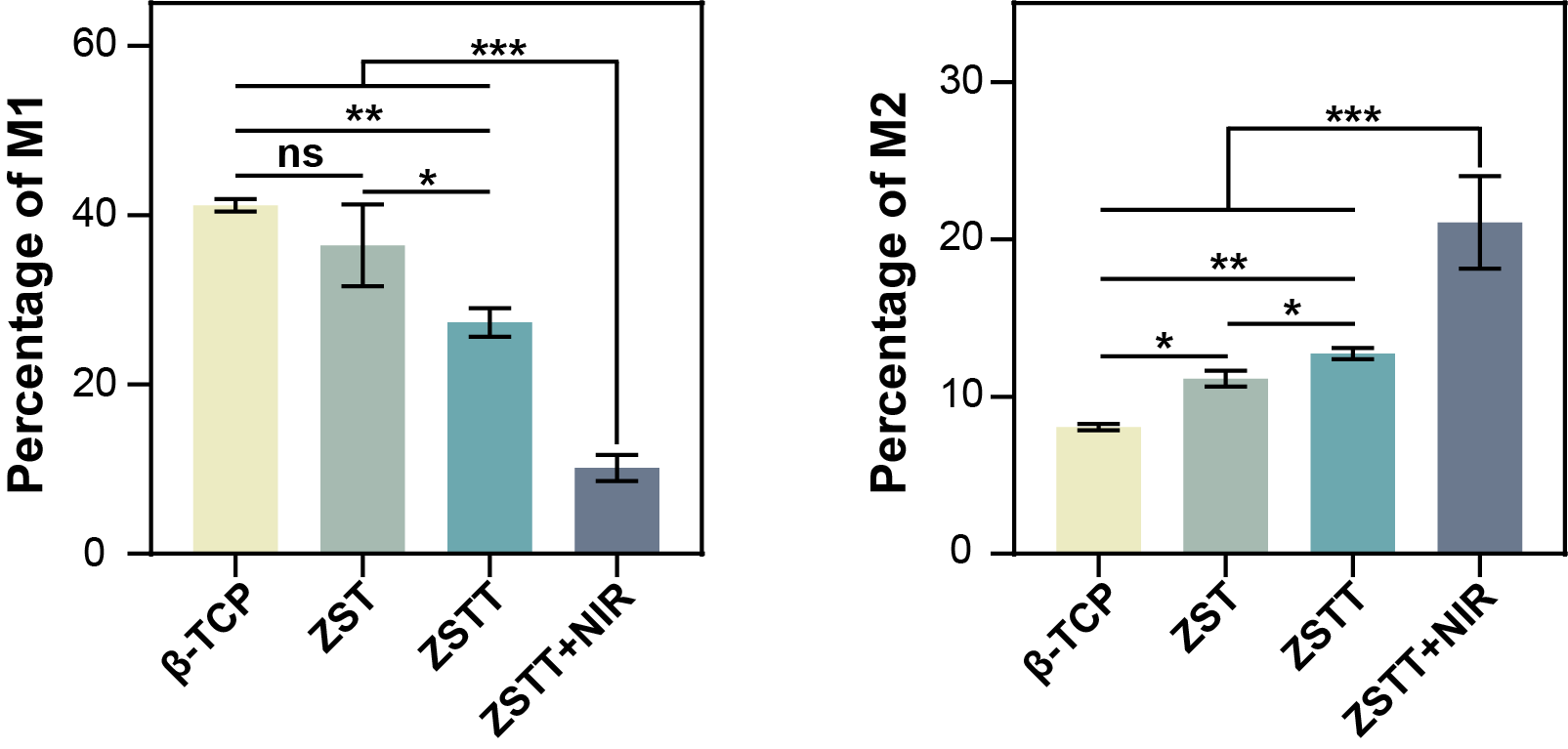


**Figure S12.** Average percentage of M1 and M2 in different groups. The data are presented as the mean ± standard deviation, n=3. (*) *p*<0.05, (**) *p*<0.01, (***) *p*<0.001, and ns: no significance.


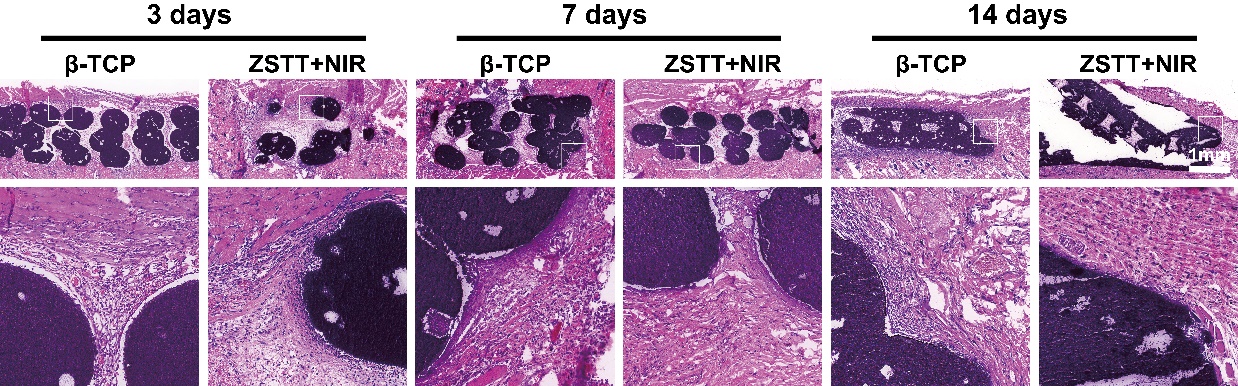


**Figure S13.** H&E staining in subcutaneous tissues around scaffolds.


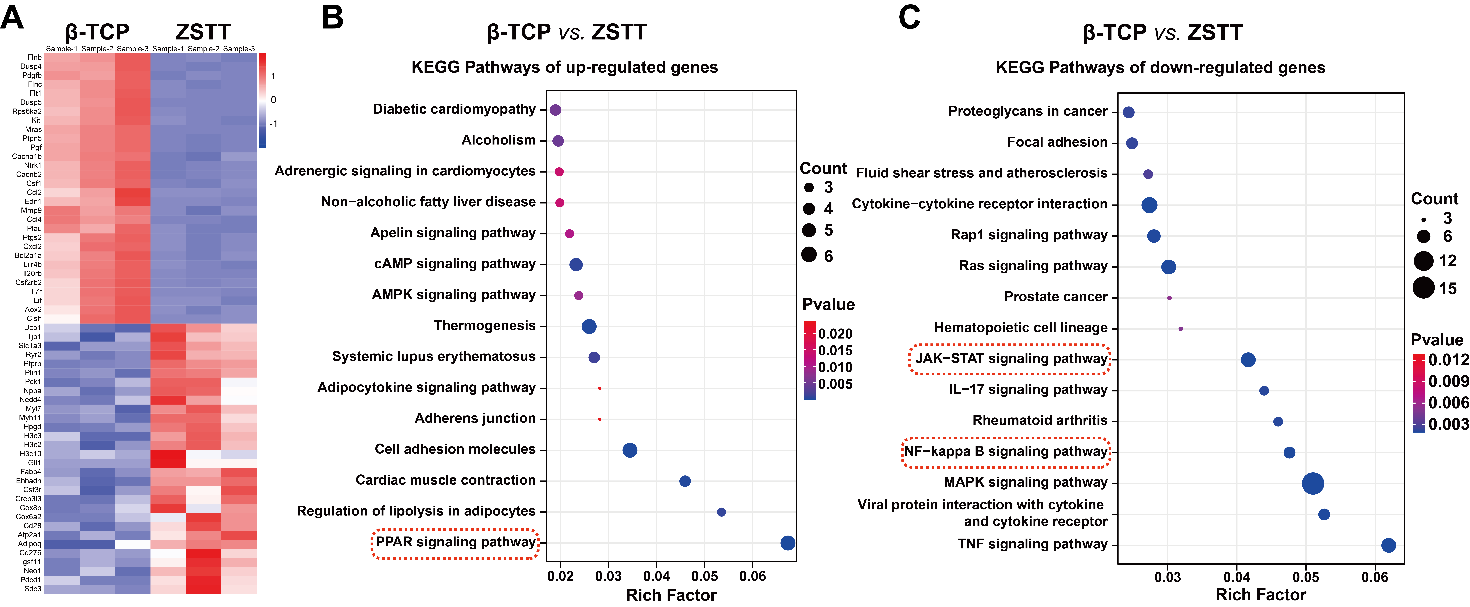


**Figure S14.** A) Heatmap of DEGs of the 60 significantly upregulated and downregulated genes of MΦs by ZSTT versus β-TCP. B&C) Enriched KEGG pathways of macrophages cultured on ZSTT versus β-TCP.


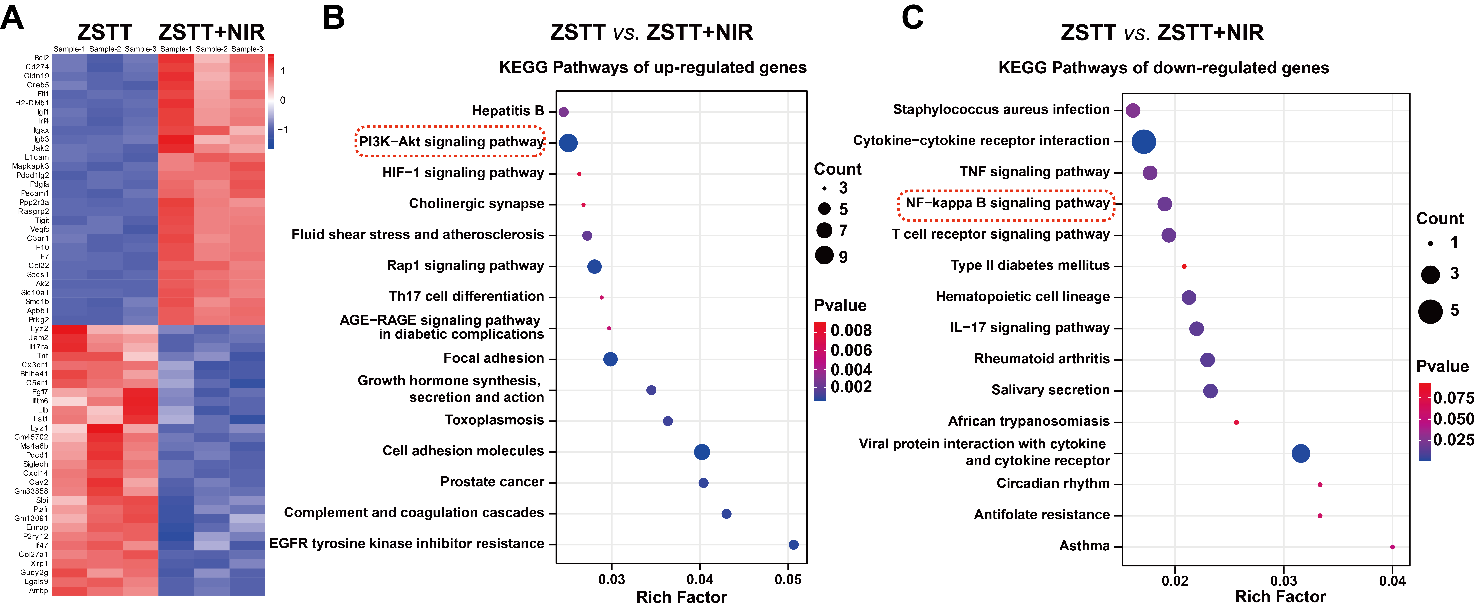


**Figure S15.** A) Heatmap of DEGs of the 60 significantly upregulated and downregulated genes of MΦs by ZSTT with NIR versus ZSTT. B&C) Enriched KEGG pathways of macrophages cultured on ZSTT with NIR versus ZSTT.


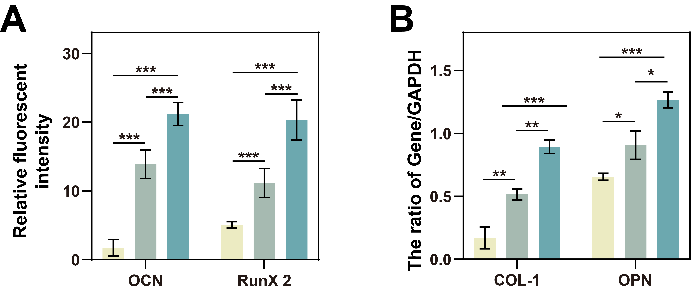


**Figure S16.** A) The corresponding quantification of Immunofluorescence for OCN and Runx2. A) The corresponding quantification of Western blotting for OPN and COL1. The data are presented as the mean ± standard deviation, n=3. (*) *p*<0.05, (**) *p*<0.01, and (***) *p*<0.001.

^[1]^


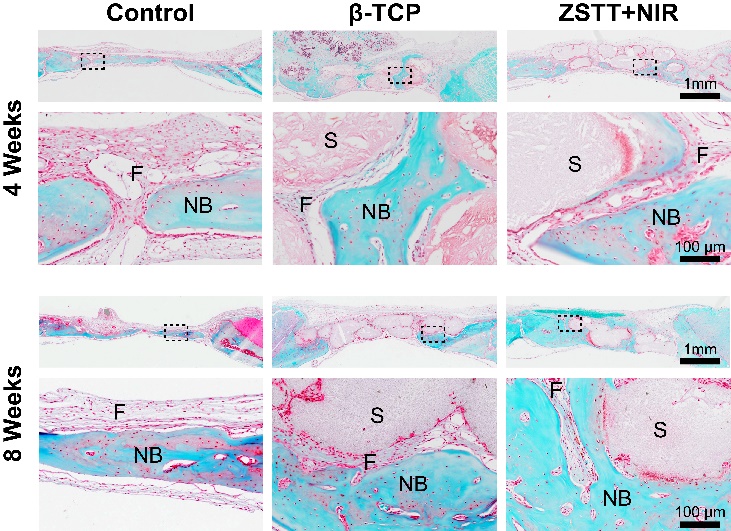


**Figure S17.** Safranin-O/Fast Green staining images at 4 and 8 weeks, including newly mineralized bone tissue (NB), fibrous tissue (F), and scaffold residuals (S).


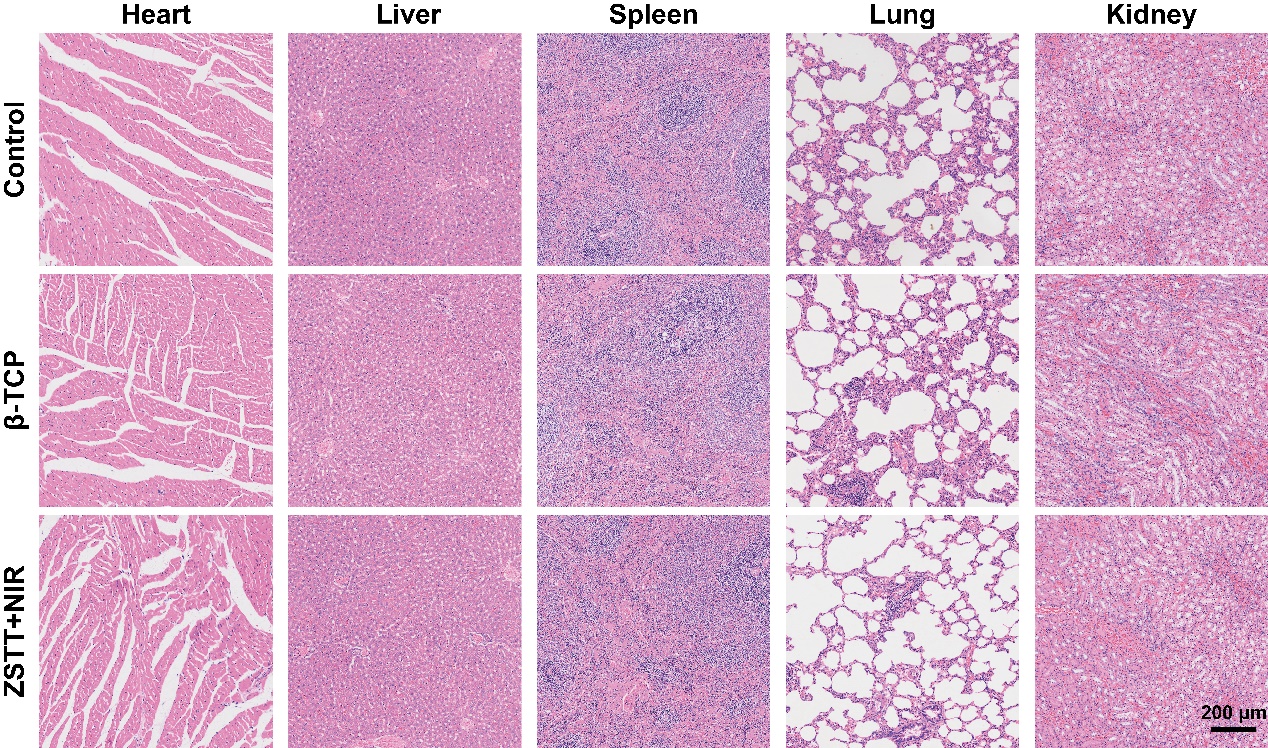


**Figure S18.** Systemic toxicity evaluation of scaffolds. H&E staining of the heart, liver, spleen, lung, and kidney across different experimental groups. n=3 independent experiments per group.

| **Table S1**. Primer sequences for qRT-PCR analysis. | | | |
| --- | --- | --- | --- |
| Gene | | Forward primer | Reverse primer |
|  | iNOS | 5'-TTTGCTCATGACATCGACCAGAA-3’ | 5'-CGTTTCGGGATCTGAATGTGATG-3’ |
|  | IL-1β | 5'-CAGCACATCAACAAGAGCTTCAG-3’ | 5'-GAGGATGGGCTCTTCTTCAAAGA-3’ |
|  | TNF-α | 5'-GCCTCCCTCTCATCAGTTCTATG-3’ | 5'-ACCTGGGAGTAGACAAGGTACAA-3’ |
|  | Arg-1 | 5'-TCTGCCAAAGACATCGTGTACAT-3’ | 5'-CGACATCAAAGCTCAGGTGAATC-3’ |
|  | IL-10 | 5'-CAGAGAAGCATGGCCCAGAAATC-3’ | 5'-GCTCCACTGCCTTGCTCTTATTT-3’ |
|  | CD206 | 5'-TCCCTGTCTCTGTTCAGCTATTG-3’ | 5'-CGTCTGAACTGAGATGGCACTTA-3’ |
|  | GAPDH | 5'-CAGTGGCAAAGTGGAGATTGTTG-3’ | 5'-TCGCTCCTGGAAGATGGTGAT-3’ |
|  | ALP | 5'-GCATAGACTTCAACCAGCCTTTG-3’ | 5'-CTCCGTACCAAAGCCATCGATAG-3’ |
|  | Runx2 | 5'-GGCCACTTACCACAGAGCTATTA-3’ | 5'-GTGTCTGCCTGGGATCTGTAATC-3’ |
|  | OPN | 5'-ATGAGTCCTTCACTGCCAGC-3’ | 5'-CATCGGACTCCTGGCTCTTC-3’ |
|  | OCN | 5'-GAGGACCCTCTCTCTGCTCA-3’ | 5'-GGTAGCGCCGGAGTCTATTC-3’ |
|  | GAPDH | 5'-GAAGGTCGGTGTGAACGGAT-3’ | 5'-CCCATTTGATGTTAGCGGGAT-3’ |
|  | AKT-1 | 5'-ATGAACGACGTAGCCATTGTG-3’ | 5'-TTGTAGCCAATAAAGGTGCCAT-3’ |
|  | PPAR-γ | 5'-GGAAGACCACTCGCATTCCTT-3’ | 5'-GTAATCAGCAACCATTGGGTCA-3’ |
|  | JAK-2 | 5'-GGAATGGCCTGCCTTACAATG-3’ | 5'-TGGCTCTATCTGCTTCACAGAAT-3’ |
|  | TNF-α | 5'-CAGGCGGTGCCTATGTCTC-3’ | 5'-CGATCACCCCGAAGTTCAGTAG-3’ |
